# Molecular Phylogeny of a RING E3 Ubiquitin Ligase, Conserved in Eukaryotic Cells and Dominated by Homologous Components, the Muskelin/RanBPM/CTLH Complex

**DOI:** 10.1371/journal.pone.0075217

**Published:** 2013-10-15

**Authors:** Ore Francis, Fujun Han, Josephine C. Adams

**Affiliations:** School of Biochemistry, University of Bristol, Bristol, United Kingdom; Ohio State University Comprehensive Cancer Center, United States of America

## Abstract

Ubiquitination is an essential post-translational modification that regulates signalling and protein turnover in eukaryotic cells. Specificity of ubiquitination is driven by ubiquitin E3 ligases, many of which remain poorly understood. One such is the mammalian muskelin/RanBP9/CTLH complex that includes eight proteins, five of which (RanBP9/RanBPM, TWA1, MAEA, Rmnd5 and muskelin), share striking similarities of domain architecture and have been implicated in regulation of cell organisation. In budding yeast, the homologous GID complex acts to down-regulate gluconeogenesis. In both complexes, Rmnd5/GID2 corresponds to a RING ubiquitin ligase. To better understand this E3 ligase system, we conducted molecular phylogenetic and sequence analyses of the related components. TWA1, Rmnd5, MAEA and WDR26 are conserved throughout all eukaryotic supergroups, albeit WDR26 was not identified in Rhizaria. RanBPM is absent from Excavates and from some sub-lineages. Armc8 and c17orf39 were represented across unikonts but in bikonts were identified only in Viridiplantae and in *O. trifallax* within alveolates. Muskelin is present only in Opisthokonts. Phylogenetic and sequence analyses of the shared LisH and CTLH domains of RanBPM, TWA1, MAEA and Rmnd5 revealed closer relationships and profiles of conserved residues between, respectively, Rmnd5 and MAEA, and RanBPM and TWA1. Rmnd5 and MAEA are also related by the presence of conserved, variant RING domains. Examination of how N- or C-terminal domain deletions alter the sub-cellular localisation of each protein in mammalian cells identified distinct contributions of the LisH domains to protein localisation or folding/stability. In conclusion, all components except muskelin are inferred to have been present in the last eukaryotic common ancestor. Diversification of this ligase complex in different eukaryotic lineages may result from the apparently fast evolution of RanBPM, differing requirements for WDR26, Armc8 or c17orf39, and the origin of muskelin in opisthokonts as a RanBPM-binding protein.

## Introduction

Ubiquitin-dependent proteolysis of proteins by the proteasome is a major cellular mechanism for rapid modulation of protein levels in eukaryotic cells, that likely has common ancestry with a prototypic system identified in Archaea [1]. The ability to degrade proteins efficiently and specifically via the Ubiquitin Proteasome Pathway (UPP) is essential for many functions of eukaryotic cells. In humans, defects in the pathway can lead to cancers and deletions of certain components are lethal [2], [3]. In the UPP, proteins to be degraded are tagged repeatedly by covalent attachment of the 76 aa protein, ubiquitin; a polymeric chain of four ubiquitins being the minimum tag for efficient degradation [4]. Ubiquitination occurs through a pathway catalyzed by ubiquitin-activating enzymes (E1), ubiquitin conjugating enzymes (E2) and ubiquitin-protein ligases (E3) [5]. Two E1 enzymes, over 35 E2 enzymes and over 600 E3 ubiquitin ligases are encoded in the human genome [4]–[6]. Many E3 enzymes are characterised by one of two protein domains that are essential for E3 ligase activity: the Homologous to the E6-AP Carboxyl (HECT) domain or the Really Interesting New Gene (RING) domain [4]. HECT E3s accept activated ubiquitin from the E2 before substrate modification, whereas RING E3s act as scaffolds that bind the substrate and the ubiquitin-loaded E2 concurrently. Specificity of the UPP for target proteins is determined by E2/E3 pairings. Additional variability is achieved by RING domain proteins that participate in multi-protein complexes, in which other proteins act as adaptors for recognition or stabilisation of interactions with the specific substrate [7], [8].

The *S. cerevisiae* glucose induced degradation deficient (GID) complex is a 600 kDa assembly of seven proteins: GID1 (aka VID30), GID2, GID4 (aka VID24), GID5 (aka VID28), GID7, GID8 and GID9 [9]. The GID complex mediates polyubiquitination of fructose-1,6-bisphosphatase (FBPase) via E3 ubiquitin ligase activity of GID2, that contains a RING domain. FBPase, a rate-controlling enzyme in gluconeogenesis, is synthesized by budding yeast cells when grown on a non-fermentable carbon source. Upon replenishment of glucose, GID4 is synthesized and binds to the rest of the GID complex via GID7 to activate GID2 E3 ligase activity, leading to FBPase1 polyubiquitination and proteosomal degradation. FBPase1 can also be degraded by vesicular trafficking to the vacuole (the Vid pathway), and GID1, GID4 and GID also function in this pathway [10], [11]. Thus, the complex acts to shift carbohydrate metabolism of *S. cerevisiae* towards glycolysis [9].

**Figure 1:**
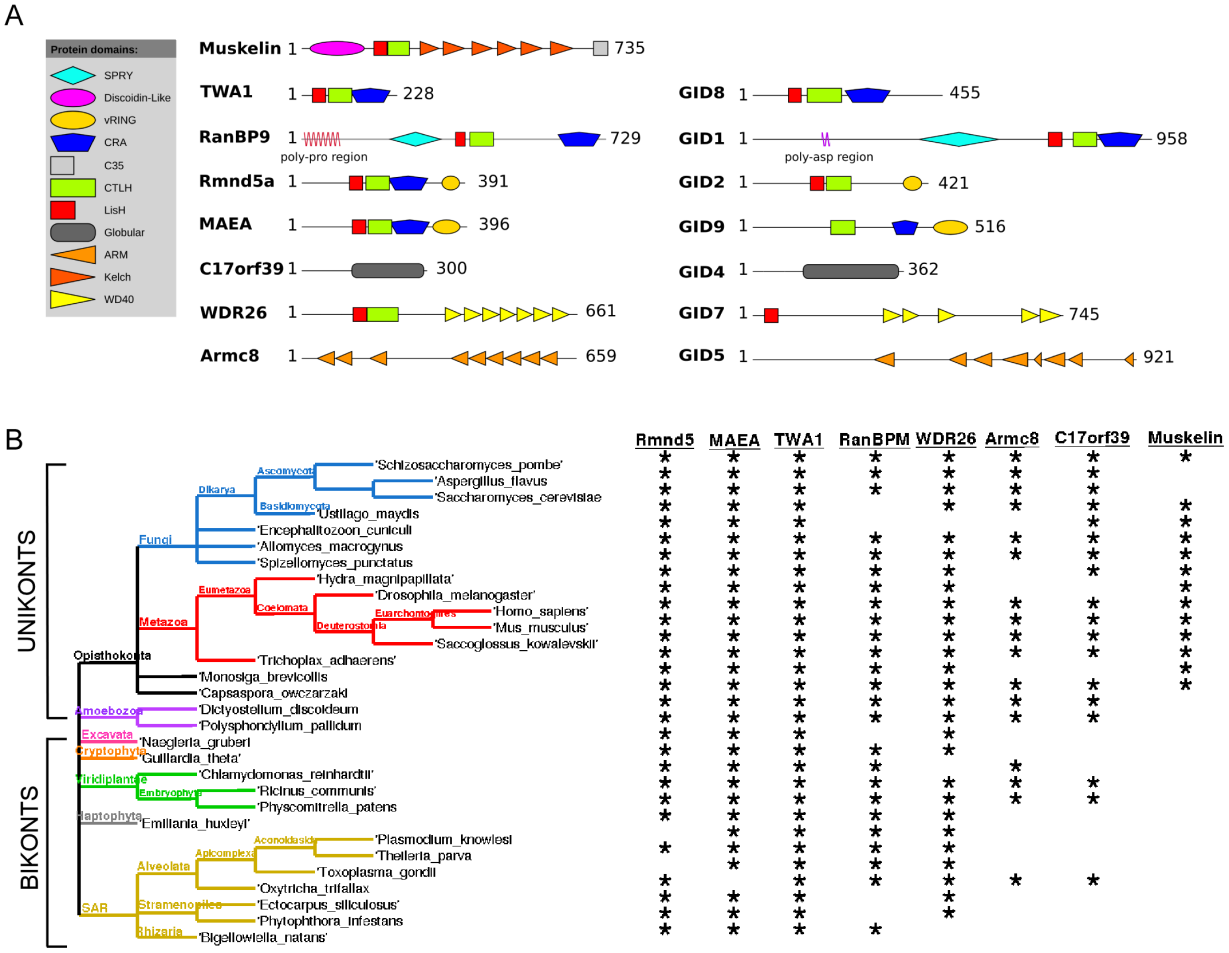
Components of the muskelin/RanBPM/CTLH complex. A, Scaled schematic diagrams of the domain organisation of muskelin/RanBPM/CTLH and GID complex proteins. Muskelin is not encoded in budding yeast. Shared domains are identified in the key. B, Muskelin/RanBPM/CTLH complex components are conserved across the domain of eukaryotes. The taxonomic tree of representative eukaryotic species is rendered in TreeDyn. Asterisks indicate the encoding of MRCTLH complex proteins in the indicated species. Absence of an MRCTLH complex proteins reflects absence of orthologues in other species of the same taxonomic lineage.

Many GID complex components have counterparts in mammals and plants. GID1, GID2, GID4, GID5, GID7, GID8 and GID9 are homologous in domain organisation and closest in sequence identity to, respectively, mammalian RanBP9 (aka RanBPM), the RING containing protein Rmnd5a (aka p44CTLH), c17orf39, ARMc8, WDR26, TWA1 (aka CT11, Bwk1 or c20orf11) and MAEA (aka HLC10, EMLP, EMP, HLC-10, PIG5) (Fig. 1). In *Arabidopsis thaliana*, a protein complex isolated by physical association with RanBPM includes homologues of TWA1, MAEA, Rmnd5 and WDR26 [12]. In mammalian cells, TWA1, Rmnd5a, MAEA, RanBP9 and Armc8 form a complex with the protein, muskelin, that is not encoded in the *S. cerevisiae* genome or in plants, and which appears to replace GID7 [13], [14]. The GID4 homologue, c17orf39, and the GID7 homologue, WDR26, were not identified within the mammalian complex [14]. Because the proximal partner of muskelin is RanBP9/RanBPM [15], [16], we refer here to the biochemically identified complex as the Muskelin/RanBP9/CTLH complex (MRCTLH).

How the mammalian complex might function as an E3 ubiquitin ligase and the roles of most components within the complex are poorly understood. ARMc8 promotes interaction of hepatocyte growth factor-regulated tyrosine kinase substrate (HRS) with ubiquitinated proteins and regulates degradation of alpha-catenin; however, ARMc8 is not necessary for assembly of the mammalian complex [17], [18]. Maea was also identified as a macrophage attachment protein of erythroblasts, with a role in nuclear extrusion in erythropoeisis [19]. In non-erythroid cells Maea is reported as a nuclear protein [20]. The complex of muskelin and RanBP9 regulates cell morphology as a function of their nucleocytoplasmic localisation; it is unknown if this activity occurs in context of TWA1, Maea or Rmnd5 [16]. RanBPM is recognised as a scaffolding protein with many potential binding partners of its N-terminal SPRY (SPla and ryanodine receptor) domain [21]–[24]. In some contexts, RanBPM levels appear to regulate the stability of other proteins [25]–[28]. MRCTLH components are all widely expressed in multiple tissues, yet MAEA, muskelin or RanBP9 knockout mice have distinct phenotypes [19], [29], [30]. In view of their potential fundamental roles in pathways of protein degradation and cellular organisation, there is a great need to better understand the relationships and roles of MRCTLH proteins.

A striking feature of this complex is that five components have similarities of domain architecture. Muskelin, RanBPM, TWA1, Maea and Rmnd5 all contain, in their central regions, LisH (for lissencephaly-1 homology) and CTLH (for C-terminal to LisH) domains, and only muskelin does not contain a following CRA (for CT11-RanBP9) domain [9], [13], [14] (Fig. 1A). LisH and CTLH domains are found in multiple proteins, some of which are associated with microtubule dynamics, nucleokinesis, cell migration and chromosome segregation [31]. As a first step to understand the nature of the GID and MRCTLH complexes in greater depth, we have examined the evolution of individual components through analyses of prokaryotic and eukaryotic genomes and phylogenetic inference. For the five components with common domains, we have further examined the sequence conservation of individual domains and compared protein localisations in cells. This comprehensive analysis leads to an improved understanding of their molecular phylogeny.

## Materials and Methods

### Database searches and dataset curation

Species orthologues of MRCTLH proteins were identified through BLASTp and TBLASTN searches with the human MRCTLH components (TWA1/Q9NWU2/NP_060366; RanBP9/Q96S59/NP_005484; MAEA/Q7L5Y9/NP_001017405; Rmnd5A/Q9H871/NP_073617; muskelin/Q9UL63/NP_037387; Armc8/NP_056211; WDR26/NP_079436; c19orf39/NP_076957) and the homologous yeast GID components (GID8/P40208/NP_013854; GID1/P53076/NP_011287; GID9/P40492/NP_012169; GID2/Q12508/NP_010541; GID5/NP_012247; GID7/NP_09891; GID4/NP_009663). Searches were run against the nonredundant databases of the National Center for Biotechnology Information (NCBI), Uniprot, and the Broad Institute, at default parameters (e value cutoff of 1e-5). The genome assemblies of the Rhizarian *Bigelowiella natans*, the haptophyte *Emiliania huxleyi* and the cryptophyte *Guillardia theta* were analysed by TBLASTN searches of filtered transcripts and ESTs at the DOE Joint Genome Institute genome portal, at default parameters. To ensure that orthologues remote from the human and yeast sequences were not missed, PSI-BLAST of GenBank proteins and TBLASTN searches of the GenBank non-redundant nucleotide, EST and GSS databases were also carried out using MRCTLH sequences from a phylogenetically diverse set of organisms, comprising *Arabidopsis thaliana*, *Aspergillus fumigatus*, *Babesia bovis*, *Dictyostelium discoideum*, *Ectocarpus siliculosus*, *Homo sapiens*, *Micromonas sp. RCC299*, *Monosiga brevicollis*, *Naegleria gruberi*, *Paramecium tetraurelia*, *Saccharomyces cerevisiae*, *Schizosaccharomyces pombe*, *Toxoplasma gondii*. More stringent e value cutoffs of 1e-15 (for PSI-BLAST) and 1e-30 (for TBLASTN) were applied to these searches to ensure that the analysis of hits from these wider search methods concentrated on likely orthologues.

Proteins identified in the original BLAST searches were then used as queries in reciprocal BLASTP or TBLASTN searches. Proteins were considered to be orthologues of the MRCTLH complex component returned with the most significant e-value [32]. This was confirmed by PhyML phylogenetic analysis of the dataset gathered for each complex component [33]. These proteins scanned for domain composition using InterProScan [34] at The Wageningen University Bioinformatics Webportal (http://www.bioinformatics.nl/iprscan/). A two domain lower limit was imposed on the sequences to be retained in the dataset. Preference was also given to retention of Reference Protein sequences in the datasets. Only the top e-value within each species was included in subsequent analyses of domains. Where components were identified to be encoded at more than one chromosomal location in the same genome, entries that represented both paralogues were retained. The dataset gathered for each complex component was also analysed by construction of PhyML phylogenetic trees from MUSCLE multiple sequence alignments of the full-length sequences, to ascertain that the identified sequences showed phylogenetic congruence within a tree. Apart from some very divergent sequences from parasites such as *E. cuniculi*, sequences identified by the BRBH criterion clustered in appropriate clades within these trees.

### Taxonomic tree construction

The taxonomic origin of each entry was determined based on the NCBI Taxonomy ID (Tax ID) database. The Tax ID numbers were used to generate taxonomic trees in PHYLIP format with the NCBI XML tree tool (http://ncbi.nlm.nih.gov/tools/cobalt/cobalt.cgi). PHYLIP files were rendered using Treedyn [35].

### Alignments and phylogenetic analysis of MRCTLH proteins with LisH and CTLH domains

Protein sequences corresponding to TWA1, RanBPM, MAEA and Rmnd5 orthologues from *Arabidopsis thaliana*, *Aspergillus fumigatus*, *Dictyostelium discoideum*, *Ectocarpus siliculosus*, *Homo sapiens*, *Monosiga brevicollis*, *Naegleria gruberi*, *Saccharomyces cerevisiae and Schizosaccharomyces pombe* were used to generate profile alignments for each domain in each protein. The predicted boundaries of the LisH and CTLH domains, as represented in the human protein sequences in each alignment and secondary structure predictions obtained in Jpred3 for each protein in the profile, were used in setting boundary criteria for all sequences within the profile. In the first alignments representation of proteins was in accordance with the species representation of each protein in the eukaryotic supergroups; however, any sequences that were incomplete for these domains were excluded from following alignments. A dataset of 922 sequences corresponding to the LisH and CTLH domains of MRCTLH proteins were analysed phylogenetically using SATe 2.0.3 [36]. Sub-alignments were made with MAFTT then merged with MUSCLE [37]. Phylogenetic trees were estimated using RAxML v 7.2.6 [38] with the CATWAGF model and all other parameters at default settings.

Taxon representation varied widely between the supergroups and orthologues in many under-represented taxa or phyla had great sequence diversity. Additional phylogenetic analyses were made based on a selected dataset of 109 sequences that represented species from all eukaryotic supergroups and available lineages within each supergroup, but included only 6–7 species orthologues for each protein from plants, animals and fungi and excluded highly divergent sequences from parasitic species. The previously identified LisH/CTLH regions were aligned in SATe, with 90 runs in total to achieve an optimal alignment using the CATWAGF model. PhyML trees were produced initially with Shimodaira-Hasegawa support values, allowing comparison of LG, WAG and BLOSUM62 amino acid substitution models. Analysis of the data with ProtTest [39] confirmed that LG was the most suitable model for these data. In addition to statistical support values, support values were derived from 1000 PhyML bootstrap trees using the LG amino acid substitution model and a rate across site variation modelled on a discrete GAMMA distribution with four rate categories. The proportion of invariable sites, the shape parameter of the distribution, and amino acid frequencies were estimated from the data. Bayesian analysis was carried out using MrBayes 3.2 [40] with the Poisson model of fixed equal amino acid substitution rates. Two runs consisting of 4 chains were run for 33000000 generations. Trees were sampled after every 1000 generations and 50% discarded for burnin. The number of states was set to 20, the rate parameter was invgamma and the shape parameter was estimated from the alignment. Chains were considered to be convergent when the average split frequency was lower than 0.01. Posterior probability values were appended to the consensus tree. The Newick outputs of all trees were rendered in integrated Tree of Life (iTOL) (itol.embl.de) [41].

### Sequence logos

Sequences that contained LisH and CTLH domains as identified in InterProScan were selected from the complete dataset of MRCTLH proteins. For sequences in which only one of the two domains was identified, a region of 50aa N-terminal to the CTLH domain was taken to correspond to the LisH domain (typical length of a LisH domain is 35 aa) [31], and a region of 70aa C-terminal to the LisH domain was taken to correspond to the CTLH domain (typical length of a CTLH domain is 50aa) [31]. To capture sequences in which LisH and CTLH domains were not defined by InterPro, all sequences from each MRCLTH complex component were segregated according to eukaryotic super-group to capture lineage-specific distinctions and aligned in MUSCLE at default settings [37] (www.ebi.ac.uk/Tools/muscle) and the sequence regions of proteins that aligned with the known LisH and CTLH domains extracted for inclusion in the Logo dataset. The MUSCLE multiple sequence alignment for each MRCTLH protein was submitted to Weblogo at default parameters to generate Sequence Logos [42] (http://weblogo.berkeley.edu). Consensus Logos for all LisH, CTLH and RING domains were prepared from the respective non-redundant sequences in the SMART database. Human and *S.cerevisiae* sequences were also analysed separately by pairwise alignments using the EMBOSS alignment tool with the Needleman-Wunsch algorithm [43] at default settings (http://www.ebi.ac.uk/Tools/emboss/align/).

### Secondary structure prediction

LisH and CTLH domain sequences from the same dataset as above were submitted to the Jpred3 server [44] via Jalview ([45]. Secondary structure predictions outputs were compared and those discussed here are from the Jnetpred profile output. Conserved residues within the LisH domain, as identified from sequence Logos, were mapped to corresponding residues of template crystal structures and rendered in Jmol (http://www.jmol.org/). The crystal structures used as templates were the LisH domain from LIS1 (PDB 1UUJ) [46] and the RING domain from the Tripartite motif protein 32 (TRIM32; PDB 2CT2).

### Expression constructs

Mammalian expression constructs for human RanBP9 were as previously described [16]. Expression constructs for human MAEA and Rmnd5a were prepared by TOPO cloning of PCR amplified cDNAs corresponding to full-length MAEA, MAEA N-terminus (aa 1–216), MAEA C-terminus (aa 159–396), Rmnd5a, Rmnd5a N-terminus (aa 1–213) or Rmnd5a C-terminus (aa157–395) into pcDNA3.1/V5-His-TOPO (Invitrogen) according to manufacturer's procedures. Expression constructs for 3×FLAG-tagged TWA1, TWA1 N-terminus (aa 1–120), or TWA1 C-terminus (aa 63–228) were prepared by subcloning of cDNAs generated with appropriate restriction enzyme sites by PCR, between the HindIII and EcoRI sites of pCMV3×FLAG (Sigma). The oligonucleotide primers used are listed in Table S1 and were synthesized by Sigma-Aldrich. All constructs were validated by DNA sequencing (Eurofins MWG). Protein expression was confirmed by immunoblots of whole cells lysates of transfected COS-7 cells with antibodies to the respective epitope tags; either FLAG (mouse monoclonal antibody clone M2; Sigma) or V5 (mouse monoclonal to V5 tag; Invitrogen).

### Immunofluorescence microscopy

2×10^5^ COS-7 cells were transfected with 2.5 µg of the relevant plasmid using Polyfect reagent (Qiagen) according to manufacturer's protocol and cultured for 12 h, then trypsinized and plated on glass coverslips in 6-well plates. 24 h later, cells were fixed in 2% (v/v) paraformaldehyde in phosphate buffered saline (PBS) for 10 min and permeabilized with 0.5% Triton X-100 in PBS for 10 min. Cells were stained as appropriate with FITC-conjugated mouse monoclonal antibody to FLAG-epitope (clone M2, Sigma-Aldrich) or FITC-conjugated goat polyclonal antibody to V5 epitope (Abcam) in PBS containing 2% BSA for 1 h, then washed and mounted in Vectashield mounting fluid containing DAPI (Vector Labs). Non-transfected cells, or cells transfected with FLAG or V5 control plasmids, were used as controls for background fluorescence. Images in *XY* and X*Z* were acquired on an inverted TCS-SP2 AOBS confocal microscope (Leica, Wetzlar, Germany) using a 63 X oil immersion objective (NA 1.40) and PhotoMultiplier Tubes (PMT), PMT1 (R6357, Hamamatsu), PMT2 (R658, Hamamatsu) and PMT3 (R658, Hamamatsu) with appropriate filter-sets. Images were captured by Leica Confocal software v2.61.

## Results

### The phylogenetic distribution of Muskelin/RanBPM/CTLH complex components within eukaryotes

The MRCTLH and GID complexes are homologous multi-protein complexes, identified in mammals and budding yeast, respectively [9], [14]. Domain organizations are conserved between the human and yeast proteins, albeit that the *S. cerevisiae* complex components GID1, GID8 and GID9 are each markedly longer than the mammalian counterparts (Fig. 1A). To gain knowledge of the phylogenetic distribution of each complex component, genome (nucleotide and predicted proteome), protein, and expressed sequence tag (EST) databases from eukaryotes and prokaryotes were searched by BLAST methods, first with the sequences of each human and *S. cerevisiae* orthologue and subsequently with sequences from representative phylogenetically diverse organisms (see Methods). Because of the overall similarity of domain organization between components such as TWA1, Rmnd5 and Maea (Fig. 1A), multiple criteria were applied for the identification of orthologous proteins in different species. These included: 1) the presence of at least 2 domains in the correct order; 2), returning a higher identity to one complex component than all others in reciprocal BLAST searches, 3), appropriate clade placement within phylogenetic trees constructed for each complex component, and 4), in most cases, having a protein sequence length similar to either the human or yeast orthologues.

No proteins with equivalent domain architectures were identified in any prokaryotes, however the discoidin domain [47], WD repeats, kelch repeats (e.g., *Vibrio cholerae*, GI: 14195570), armadillo repeats and LisH domain are present in both bacteria and archaea, and the CTLH and CRA domains are present in bacteria. *P. pallidum* MAEA contains a predicted domain between aa27–119, DUF342, that exists primarily in bacteria (Pfam: PF03961). The curated list of eukaryotes identified to encode MRCTLH proteins includes 312 species spanning all eukaryotic supergroups (see taxonomic trees, Figure S2). 106 Metazoa, 1 independent opisthokont, 2 choanoflagellates, 145 Fungi, 23 Viridiplantae, 20 alveolates, 4 stramenpiles, 1 Rhizaria, 4 Amoebozoa, 1 Excavate, 2 Cryptophytes and 1 Haptophyte species were represented, reflecting the current taxonomical sampling imbalance of species selected for genome projects. Overall, the analysis identified that the phylogenetic distribution of Muskelin is restricted to opisthokonts; muskelin is also present in lower numbers of fungal species than the other proteins (Fig. 1B, Figure S1) [48]. Rmnd5, MAEA, TWA1 and WDR26 were revealed to each be distributed in all eukaryotic supergroups, with apparent loss of WDR26 from Rhizaria (Fig. 1B and Figure S1). RanBPM was represented within all unikont supergroups and in the bikont Viridiplantae, Chromalveolates and the crypotophyte and haptophyte species, but was not identified in Excavates, the stramenopiles lineage of Chromalveolates or microsporidian fungi (Fig. 1B and Figure S1). Armc8 and c17orf39 were distributed across unikonts but in bikonts were identified only in Viridiplantae and in *O. trifallax* within alveolates (Fig. 1B and Figure S1).

In most species, a single coding sequence was identified as the orthologue for each component, however gene duplications were noted in some plant species (Table 1). Gene duplications are also evident in the vertebrate lineage. Vertebrates encode two RanBPM paralogues, designated RanBP9 and RanBP10, which have around 58% protein sequence identity [24]. Because only RanBP9 has been identified in the mammalian complex, we focused on this paralogue. Vertebrates also encode two Rmnd5 paralogues, Rmnd5a and Rmnd5b, (70% identical in human). Only Rmnd5a has been identified in the mammalian complex [14]. The TWA1 gene is duplicated in *Xenopus leavis* and some bony fish species (e.g., NP_001090234.1 and NP_001086830.1 of *X. laevis*). Multiple coding sequences for various components were also identified in fungal species, however it was uncertain whether these represent separate gene products or polymorphisms because gene location information was not available in most cases.

**Table 1:** Plant species with multiple genes encoding MRCTLH proteins.

Species	TWA1	RANBPM	RMND5	MAEA	WDR26	ARMC8	c17ORF39
*Arabidopsis lyrata subsp. lyrata*	2	3	3	1	4	1	0
*Arabidopsis thaliana*	3	5	3	1	3	1	1
*Brachypodium distachyon*	2	1	2	1	2	1	1
*Chlamydomonas reinhardtii*	1	2	1	1	0	1	0
*Glycine max*	5	3	4	2	9	3	2
*Medicago truncatula*	1	3	3	1	2	1	1
*Micromonas sp. RCC299*	1	1	1	0	1	0	0
*Oryza sativa Japonica Group*	2	0	1	1	1	0	2
*Physcomitrella patens subsp. patens*	5	4	5	2	5	2	1
*Populus trichocarpa*	4	2	4	1	5	1	1
*Ricinus communis*	2	1	2	1	2	1	1
*Selaginella moellendorffii*	2	2	1	4	3	2	1
*Sorghum bicolor*	2	1	1	1	1	1	1
*Vitis vinifera*	2	2	1	1	5	1	1
*Volvox carteri f. nagariensis*	1	3	1	1	1	0	1

The numbers of genes encoding paralogues of each protein are indicated (source: NCBI). Muskelin is specific to opisthokonts.

The most widely conserved components, Rmnd5, TWA1 and MAEA, were also the most highly conserved with regard to sequence identities. TWA1 orthologues were the most consistent in polypeptide length. *S. cerevisiae* GID8 (TWA1), GID1 (RanBPM), and GID9 (MAEA), were each noted to be markedly longer than the average for these proteins, whereas the human orthologues were close to the mean length (see protein lengths in Fig. 1A). Notably, the sequences of all the human MRCTLH proteins are closer to the overall eukaryotic consensus sequences for each protein than the *S. cerevisiae* homologues. For example, GID8 is 14% identical to human TWA1, whereas *N. gruberi* TWA1 is 36.6% identical to human TWA1. RanBPM homologues were less conserved in terms of sequence identity, and varied in length from 500–900 aa. Most RanBPM homologues lacked the N-terminal poly-proline domain identified in the human and mouse proteins. RanBPM homologues in some fungi, alveolates, amoebozoa, *Ricinus communis*, and some Diptheran species have serine-rich regions near the N-terminus. The N-terminal region of *Monodelphis domestica* RanBPM is poly-glutamate-rich, and the N-terminal region of *S. cerevisiae*, *Zygosaccharomyces rouxii* and *Ashbya gossipya* RanBPMs are rich in aspartic acid residues. We also identified Apicomplexa proteins that have an Rmnd5-like RING domain but do not share any of the other domains of Rmnd5 (e.g., *Toxoplasma gondii* XP_002366075) and proteins from stramenpiles that contain a RanBPM-like SPRY domain but lack the other characteristic domains of RanBPM (e.g. *Ectocarpus silculosus* CBJ29317). Because of the lack of LisH/CTLH domains these were not entered into the dataset.

### Phylogenetic relationships of TWA1, MAEA, Rmnd5 and RanBPM

To investigate the phylogenetic relationships of TWA1, MAEA, Rmnd5 and RanBPM that have multiple shared domains, we conducted phylogenetic inference analyses based on the shared LisH/CTLH domains (around 100aa in length). A first analysis was made by the maximum likelihood method, PhyML, with the full dataset of 922 sequences that included the muskelin orthologues. Most RanBPM sequences formed a distinct cluster within the TWA1 clade, suggesting a close relationship of the LisH/CTLH regions of these two proteins (Figure S2). The LisH/CTLH regions of Rmnd5 and MAEA each formed distinct clades, yet were more closely related to each other than to RanBPM or TWA1 (Figure S2). Although the taxonomical data demonstrate much later evolutionary origin of muskelin than the other components (Fig. 1B and Figure S1), however, the majority of muskelin LisH/CTLH sequences formed a sub-cluster within the MAEA group (Figure S2). This apparent relationship should be interpreted with caution because of the known later origin and smaller number of muskelin sequences within the dataset. For each set of orthologues, some sequences located outside their main cluster; excluding muskelin, this represented 22 of the 922 sequences (2.4%).

**Figure 2:**
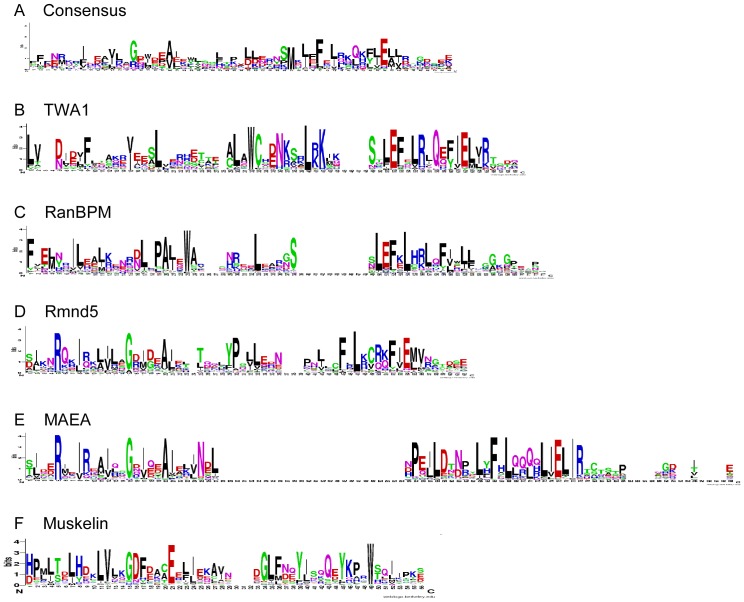
Phylogenetic relationships of RanBPM, TWA1, MAEA and Rmnd5, based on their LisH/CTLH regions. 109 sequences spanning the LisH and CTLH domains of these proteins from species representing all the eukaryotic lineages in which each of these MRCTLH protein is distributed, but excluding parasitic species (100 aa; species orthologues in the dataset are identified in Figure S4) were aligned in Sate via MAFFT and MUSCLE. Phylogenetic trees were constructed in PhyML (A) or Mr. Bayes (B) and are presented as unrooted trees with proportionate branch lengths. In A and B, the clade for each protein is labelled. Scale bars indicate substitutions/site. In A, branch support values are bootstrap values from 1000 cycles/SH ratios; in B, figures are posterior probability values.

Because the tree topology from this large dataset might be influenced by the prevalence of sequences from metazoa, plants and fungi, further phylogenetic analyses were carried out with a dataset of 109 sequences that included balanced representation of MAEA, RanBPM, Rmnd5 and TWA1 orthologues from species from all eukaryotic supergroups and available lineages within each supergroup. Muskelin was excluded from these analyses because of its later evolutionary origin (Fig. 1B and Figure S1). Highly divergent sequences from parasitic species were also excluded. Trees prepared by the maximum likelihood method, PhyML, demonstrated closer sequence relationships of Rmnd5 and MAEA, or RanBPM and TWA1, respectively. Indeed, MAEA did not form a monophylogenetic clade with respect to Rmnd5 (Fig. 2A). However, most branches in the tree had low bootstrap support, perhaps unsurprisingly given the short sequences used to build the tree and the inherent diversity of the sequences, as indicated by branch lengths, especially within the MAEA and RanBPM clades. Tree-building by Mr. Bayes also identified the closer relationships of Rmnd5 and MAEA, or RanBPM and TWA1, respectively, although clade support was weak, with most posterior probability values <0.9. In the Baysian tree, RanBPM was not monophylogenetic with respect to TWA1. Thus, although the segregation of TWA1/RanBPM versus MAEA/Rmnd5 was consistently retrieved in different trees, the phylogenetic relationships of the four proteins could not be resolved unambiguously (Fig. 2B).

### Analysis of LisH domains

To better understand the relationships between the LisH/CTLH regions, we examined the sequence conservation of the LisH and CTLH domains in detail. Because the LisH and CTLH domains are common features of five MRCTLH complex components, they have been suggested to have central roles in the assembly of the complex [14]. The LisH domain has been intensively studied in the Lissencephaly-1 (Lis1) protein, where it mediates formation of anti-parallel dimers [31], [46]. LisH dimerisation activity has also been documented in the FGFR1OP protein [49]. However, previous experiments by this laboratory identified that the LisH domain of muskelin contains unusual charged residues and functions as a regulated nuclear localisation motif [16]. Thus these domains might be specialised for different functions in different protein contexts.

**Figure 3:**
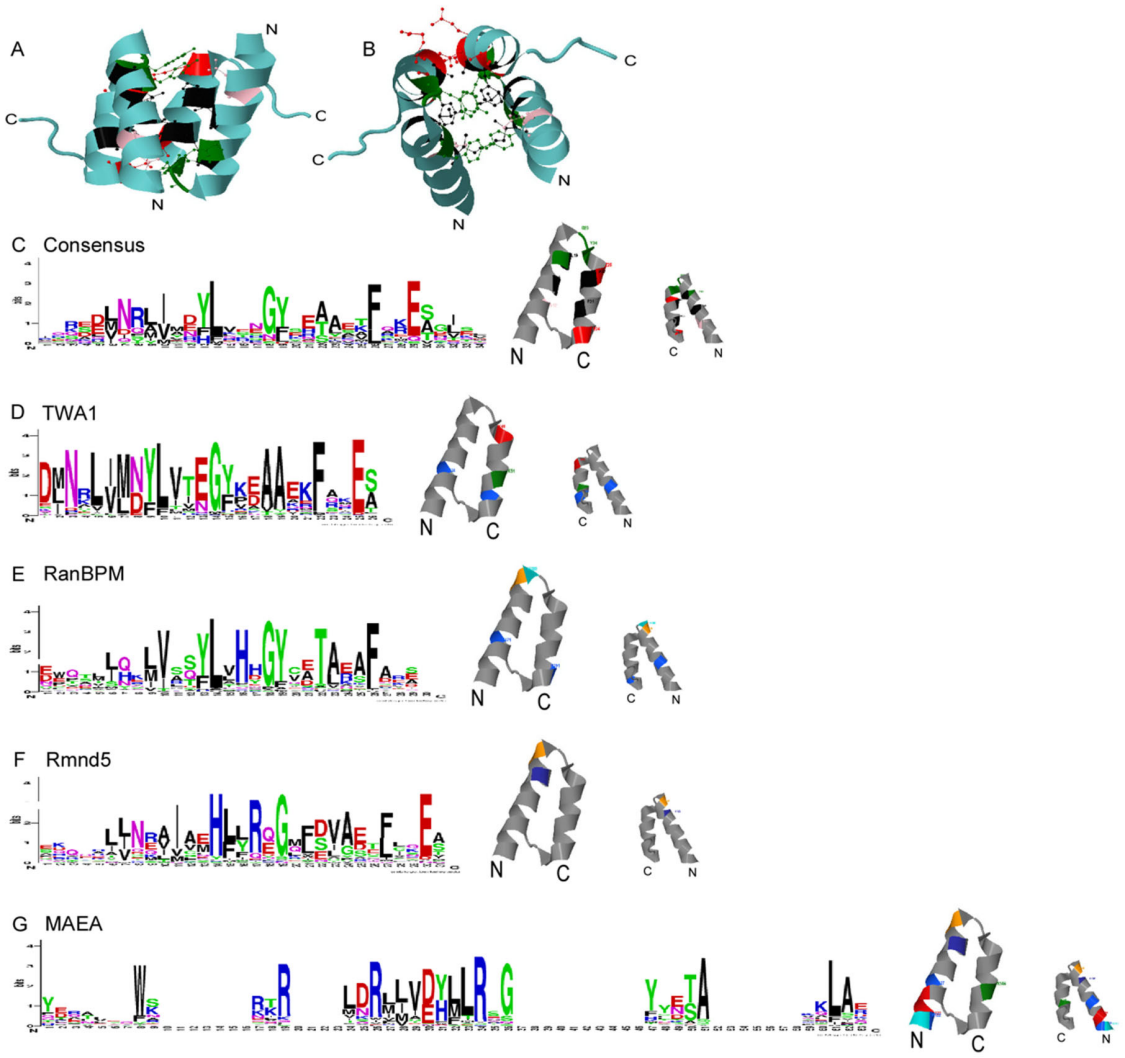
Analysis of the LisH domains of RanBPM, TWA1, MAEA and Rmnd5 by Sequence Logos. A, B, structure of the LisH domain of Lis1. Structural diagrams show side (A) and top (B) views of the two LisH domains that form an antiparallel dimer in human Lis1 (PDB 1UUJ). C, Sequence Logo consensus of the LisH domain, derived from all LisH domains in the SMART database. Amino acids are in single letter code with basic residues in blue, acidic residues in red, hydrophobic residues in black, polar residues in green, and amide-containing residues in pink. Asterisks indicate the most highly-conserved residues. The adjacent side views of a single LisH domain from Lis1 (lefthand, showing inner face, righthand showing outer face according to the dimer structure) identify the positions of these residues in colours corresponding to the Logo. D–G, Sequence Logos for the LisH domain of the indicated MRCTLH complex proteins. For each Logo, the positions of positively charged residues (blue) and negatively charged residues (red) with bit scores ≥2 are mapped onto the structure of a single LisH domain from Lis1 (lefthand shows inner face, righthand shows outer face according to the dimer structure).

We analysed consensus sequences and predicted structural features of the LisH domains of TWA1, RanBPM, Rmnd5 and MAEA with reference to the structure of the LisH domain of Lis1 [46], and to a consensus sequence Logo based on all LisH domains in the SMART database. The LisH domain of Lis1 consists of two α-helices in which the N-terminal α-helix is longer than the C-terminal α-helix (Fig. 3A). The helices are connected at the apex by a highly-conserved loop, G23Y24 (in green in Fig. 3A). In Lis1, residues 12, 15, 18 and 19 of the LisH domain are located on the first α-helix and face towards the second α-helix. In the second α-helix, residues 26, 27, 31 and 34 face towards the first α-helix (Fig. 3A). The domain forms a *trans* anti-parallel dimer, in which these strongly conserved residues form a helix interface and thus a hydrophobic core (Fig. 3B) [46]. The consensus LisH Logo shows that the well-conserved positions, likely involved in dimerisation, are uncharged residues at Logo positions 7, 10, 13, 14, 18, 19, 22 and 26 and glutamate at position 29 (asterisks in Fig. 3C). Thus, the consensus Logo is in good agreement with the LisH domain as originally defined [31].

With regard to secondary structure, all LisH domains of human and *S. cerevisiae* MRCTLH components are also predicted to contain two α-helices separated by a loop of variable length (data not shown). The sequence Logos of each LisH domain revealed clear differences of sequence conservation in each protein. The LisH domain of TWA1 is the most similar to the LisH consensus (Fig. 3D). G17 (the equivalent of G18 in the consensus sequence Logo) is strongly conserved in TWA1 and is well-conserved in the other LisH domains, probably due to its position at the “hinge” between the two α-helices (Fig. 3D–G). In RanBPM, two unusual H residues at the C-end of the first α-helix are well-conserved. When modelled against the Lis1 structure, these residues are predicted to be at the upper end of the N-terminal α-helix (Fig. 3E). The region corresponding to the predicted second α-helix of the domain is more similar to the LisH consensus (Fig. 3E).

The Logos for the LisH domains of Rmnd5 and MAEA are the most remote from the LisH consensus. In Rmnd5, residues corresponding to Logo positions Y13, L14 and Y19 of the LisH consensus are conserved weakly if at all. Positions corresponding to residues 13 and 16 of the consensus Logo contain highly conserved, positively charged residues. An aspartic acid and two glutamates are conserved in the C-terminal α-helix (Fig. 3F). The LisH domain of MAEA is even more divergent with multiple arginines and a histidine residue conserved in the N-terminal α-helix and only hydrophobic residues highly conserved along the C-terminal α-helix (Fig. 3G). The conserved charged residues in Rmnd5 and MAEA are all predicted to lie on the N-terminal α-helix, facing away from the hydrophobic core of the dimer interface (Fig. 3F, 3G). Similarities of the LisH domains of Rmnd5 and MAEA with the LisH domain of muskelin include a lack of conservation of the YL and F residues that are strongly conserved at positions 13, 14 and 26, respectively, in the consensus LisH Logo, and strong conservation of histidine and other charged residues with the N-terminal α-helix (Fig. 3F, 3G) and [16].

### Analysis of CTLH domains

**Figure 4:**
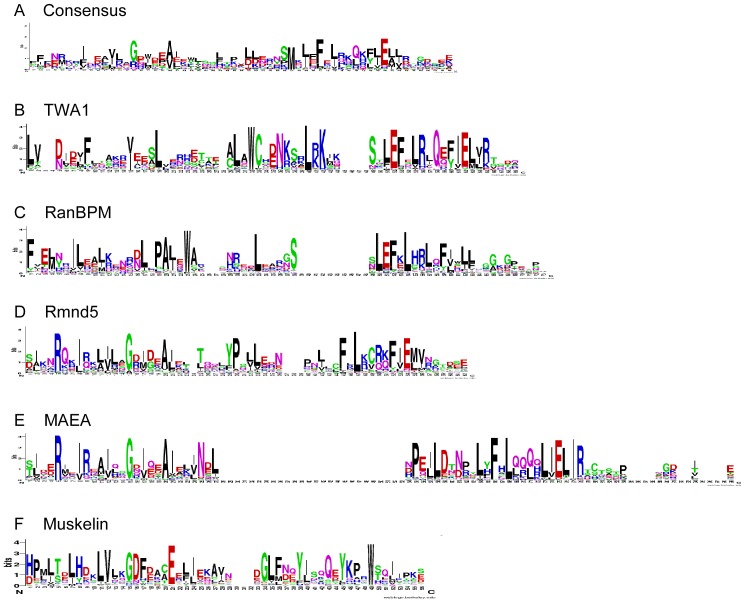
Analysis of the CTLH domains of RanBPM, TWA1, MAEA and Rmnd5 proteins by Sequence Logos. A, The Sequence Logo consensus for CTLH domains, derived from all CTLH domains in the SMART database. B–F, Sequence Logos corresponding to the CTLH domain of the indicated MRCTLH complex proteins. Colour coding as in Fig. 3.

The CTLH domains of MAEA and Rmnd5a are predicted to consist of 3 α-helices, separated by short loops. This secondary structure is equivalent to that of a consensus CTLH domain [31]. The secondary structures of the CTLH domains of RanBPM and TWA1 are predicted to consist of four α-helices separated by short loops (data not shown). Clear α-helices were not predicted for muskelin. The sequence Logos identified distinctions between the CTLH domains of the five MRCTLH components in comparison to the consensus Logo for the CTLH domain (Fig. 4). In the consensus Logo, the only well-conserved charged residue is a glutamate at Logo position 50 (Fig. 4A). An equivalent glutamate is conserved in TWA1, RanBPM, and MAEA but not in Rmnd5 or muskelin (Fig. 4). TWA1 and RanBPM contain highly conserved arginine residues in the N-terminal half of the domain (Fig. 4B, 4C). RanBPM and MAEA contain additional, highly conserved, charged residues in the C-terminal half of their CTLH domains (Fig. 4C, 4E). Highly conserved residues in the consensus Logo, G15 and A20, are strongly conserved in TWA1 and RanBPM. A glycine equivalent to G15 is conserved in muskelin (Fig. 4A vs Fig. 4B, 4C, 4F). The conservation of L38, S43, L45, E46, F47 and a tryptophan in the second α-helix further distinguishes the CTLH domains of MAEA and Rmnd5 from those of TWA1, RanBP9 and the consensus Logo. The muskelin CTLH domain is distinctive in containing conserved histidine residues in its N-terminal region, a highly conserved glutamate at Logo position 23, and lacks any strongly conserved glutamate in the C-terminal region (Fig. 4F).

### Differential roles of the LisH domains in protein localisation

**Figure 5:**
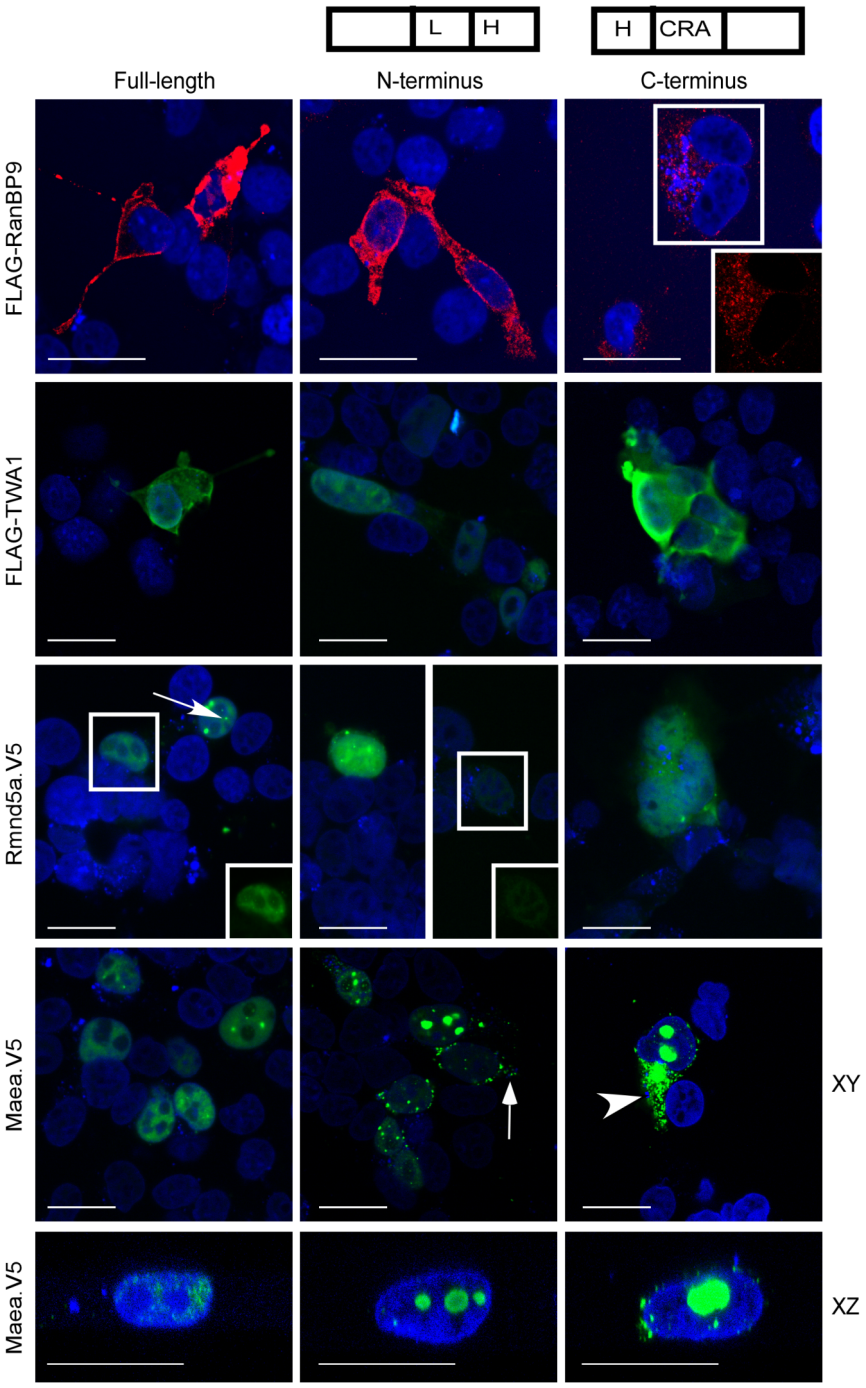
Comparison of subcellular localisations of RanBPM, TWA1, MAEA and Rmnd5. Localisations of epitope-tagged RanBP9, TWA1, Rmnd5a or MAEA and their N- and C-terminal deletion proteins (in the schematics, L = LisH domain, H = CTLH domain) were visualised in fixed COS-7 cells by immunofluorescence and laser scanning confocal microscopy. Images are in the XY plane unless otherwise stated. The merged images show each protein (in green or red) and DAPI-stained nuclei (in blue). Insets of the boxed areas show, for clarity, the corresponding single channel images for RanBP9 C-terminal protein, full-length Rmnd5a, or Rmnd5a N-terminal protein. Arrow in the image of Rmnd5a indicates small nuclear particles; arrow in the image of MAEA N-terminal protein indicates small cytoplasmic particles; arrowhead in image of MAEA C-terminal protein indicates cytoplasmic granules. Cells shown are representative of three independent experiments. Bars = 25 µm.

A positively-charged motif in the LisH domain of muskelin has a major role in controlling its nucleocytoplasmic distribution [13]. These data, and the identification of distinct sequence features in the LisH and CTLH domains of RanBPM, TWA1, Rmnd5 and MAEA (Figs. 2, 3, 4), suggested that the LisH or CTLH domains of these proteins might act to modulate their sub-cellular localisations. We therefore ectopically expressed each full-length protein in comparison with matched N- or C-terminal deletion mutants that either contained or removed the LisH domain (see domain schematics in Fig. 5). Proteins of the expected molecular masses were detected in whole cell extracts; it was notable that Rmnd5a protein was present at a lower level than MAEA protein according to detection with anti-V5 tag antibody on the same blot (Figure S3). Representative cell images from three independent microscopy experiments are shown in Fig. 5. Equivalent results were obtained in CHO cells (FH and JCA, unpublished data). RanBP9 was diffusely cytoplasmic, with a low level of nuclear fluorescence in some cells (Fig. 5, line 1). This distribution is equivalent to that of endogenous RanBP9 [16]. RanBP9 N-terminus was also diffuse throughout the cytoplasm. We reported previously that ectopic RanBP9 C-terminus accumulates in cells at much lower levels than full-length RanBP9 as determined by immunoblotting, possibly due to mis-folding or decreased stability [16]. RanBP9 C-terminus localised as small particles in the cytoplasm with apparent absence from nuclei (Fig. 5, top line). Thus, the presence of the LisH domain appears necessary for correct folding or stability of RanBP9 within the cytoplasm. TWA1 and its domain deletions are each below the size threshold for diffusion through nuclear pores [50], and indeed TWA1 and TWA1 C-terminus were distributed uniformly between the nucleus and cytoplasm (Fig. 5, line 2). TWA1 N-terminus was also detected in the nucleus and cytoplasm (Fig. 5, line 2).

The fluorescence intensity of full-length Rmnd5a was lower than the other proteins, likely due to its lower protein levels (Figure S3). Rmnd5a, of predicted molecular mass 44 kDa, was concentrated in the nucleus but was not apparent in nucleoli (Fig. 5, line 3). Most cells had diffuse nucleoplasmic fluorescence, but some cells showed small particles (example arrowed in Fig. 5, line 3). Rmnd5a N-terminus had a similar distribution, however Rmnd5a C-terminal protein was diffusely located in both cytoplasm and nucleus (Fig. 5, line 3). Given that Rmnd5a N-terminal protein has a molecular mass below the threshold for diffusion through nuclear pores [50], the results implicate the LisH domain in driving preferential nuclear localisation. Similar to Rmnd5a, MAEA, of predicted molecular mass 45.3 kDa, was located in the nucleoplasm (Fig. 5, line 4; confirmed by XZ sections as in Fig. 5, line 5) and some cells showed small nuclear particles. MAEA N-terminus (predicted molecular mass 22 kDa) was also mostly nuclear yet was much more prominent as nuclear particles than full-length MAEA (Fig. 5, line 4, and XZ section in line 5) and was detected at low level in the cytoplasm of some cells (arrowed, Fig. 5, line 4). In sharp contrast, MAEA C-terminus (predicted molecular mass 23 kDa) formed larger, granule-like aggregates within the cytoplasm (arrowhead, Fig. 5, line 4) and was restricted to nucleoli within the nucleus (Fig. 5, line 4, and XZ section in line 5). Full-length MAEA is within the size threshold for diffusion through nuclear pores, the major localisation of full-length MAEA in nuclei appears to involve both the N-terminal LisH domain and the C-terminal domains, and all domains may be needed for correct folding.

### The CRA domain

The CRA domain was identified as a 100 aa region at the C-terminus of RanBP9 that contains 6 α-helical regions and is conserved between RanBP9 and TWA1 [22]. It is now appreciated that the CRA domain is also present in MAEA and Rmnd5 (Fig. 1) [9]. Our studies demonstrate the conservation of this domain throughout eukaryotes due to its presence in multiple MRCTLH components (cf Fig. 1). Secondary structure predictions confirm that the region is largely α-helical (data not shown). No structures are available for this domain.

### Variant RING domains are shared conserved features of Rmnd5 and MAEA

**Figure 6:**
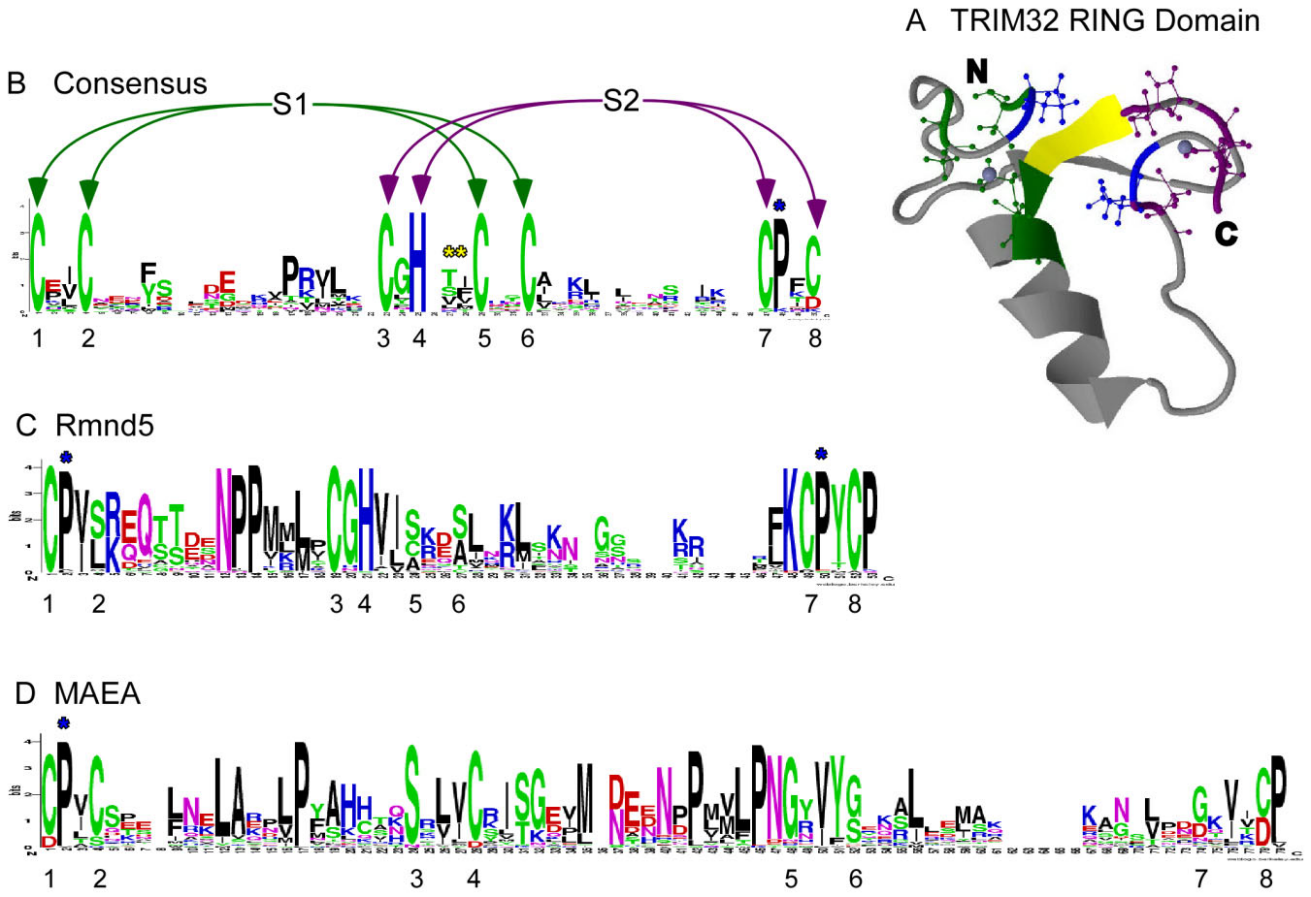
Analysis of the RING domains of Rmnd5 and MAEA. A, TRIM32 RING domain structure (PDB 2CT2). The 8 zinc-coordinating residues are shaded green or purple to distinguish between the zinc-coordinating sites S1 or S2, respectively. S1 and S2 are linked by two residues (yellow). Strongly conserved proline residues adjacent to cysteine residues of the RING domains are marked with a blue asterisk. B, consensus sequence Logo of RING domains from the SMART database. Green or purple arrows indicate the zinc-coordinating residues of sites S1 or S2, respectively. Yellow asterisks indicate residues linking S1 and S2. C, D, Sequence Logos of RING domains from Rmnd5 [C] or MAEA (D) orthologues. In all Logos, residues corresponding to the 8 zinc-coordinating residues are numbered as in 6B. Blue asterisks in C and D indicate strongly conserved prolines at the equivalent position to the well-conserved proline of the TRIM32 RING domain. The three residue gap in the Rmnd5 RING Logo is due to the extended sequence of *C. briggsae* Rmnd5 RING domain.

Rmnd5 homologues contain an additional conserved domain, a putative RING domain. BLAST searches of PDB retrieved the RING domain of TRIM32 (Fig. 6A) as the most related to the RING domain of Rmnd5a. In general, RING domains are characterised by eight conserved cysteine and histidine residues (Cx2CX(9-39)Cx(1-3)Hx(2-3)Cx2Cx(4-48)Cx2C) which adopt a cross brace motif (Fig. 6B). TRIM32 represents the RING-HC subgroup, in which cysteines at positions 1, 2, 5, and 6 of the eight conserved residues constitute the active site S1 (green residues in Fig. 6A and as marked in Fig. 6B), and three cysteines and a histidine residue at positions 3, 4, 7 and 8 constitute the second active site S2 (purple residues in Fig. 6A and as marked in Fig. 6B) [4]. Each active site coordinates one zinc ion (blue spheres in Fig. 6A). A sequence Logo for the Rmnd5 RING domain demonstrates that 3 cysteines and a histidine are conserved at positions in the Logo that correspond to S2. A single cysteine residue at Logo position 1, and serines that are the most highly conserved residues at Logo positions 4, 24 and 27, corresponds to active site S1 (Fig. 6C). Serines or glycines within the active site consensus are features of known variants of functional RING domains [51]. The Rmnd5 RING domain profile resembles that of an S/T RING domain and suggests that Rmnd5 coordinates one zinc ion only. In the TRIM32 RING domain, the S1 and S2 active sites are positioned opposite each other, separated by T40 and I41 (marked in yellow in Fig. 6A). Immediately following active site S2 position 7, a proline is strongly conserved in the RING consensus Logo (in blue in Fig. 6A, and asterisk in Fig. 6B). Proline is also conserved in this position in the Rmnd5 consensus Logo (asterisk in Fig. 6C). Strongly conserved prolines at Logo positions 2, 13, 14 and 42 (Fig. 6C) are additional notable features of the Rmnd5 RING domain sequence Logo.

Our InterPro domain analyses of MAEA from many eukaryotes identified that some orthologues, e.g., from *Polyspondiyium pallidum*, include a RING-like domain in their C-terminal regions. These regions are typically longer than the conventional RING domain and BLAST searches of PDB with the MAEA RING-like domain retrieved no hits below the 1e-5 expectation value cutoff. To investigate this further, the C-terminal regions of all MAEA sequences in our dataset were aligned and then analysed by sequence Logo. A distinct Logo profile was returned (Fig. 6D). Well-conserved cysteines were present only at Logo positions 1 and 4 (corresponding to positions 1 and 2 of the S1 active site consensus) and Logo positions 27 and 66 (corresponding to positions 4 and 8 of the S2 active site consensus). This suggests that zinc ions are not chelated by MAEA. However, serine is the most conserved residue at Logo position 23 (corresponding to position 3 of S2) and glycine is the most conserved residue at Logo position 46, 50 and 62 (corresponding to positions 5, 6 of S1 and position 7 of S2; Fig. 6D). This profile includes features of both the RING-S/T and RING-G variants [51]. Similar to Rmnd5, the MAEA Logo is distinguished from the general RING consensus Logo by the presence of strongly conserved proline residues at Logo positions 2, 16, 40, 44 and 67 (Fig. 6D, asterisk at position 2). Although conservation of proline at Logo position 2 is not apparent in the general RING consensus (Fig. 6B), multiple well-characterised RING proteins contain a proline at position 2 that is required for E3 ligase activity; for example, CNOT1 [52] or PML [53]. Overall, our sequence analyses identify that both MAEA and Rmnd5 contain conserved RING domains. This provides further evidence for a shared evolutionary origin of these two proteins.

## Discussion

**Figure 7:**
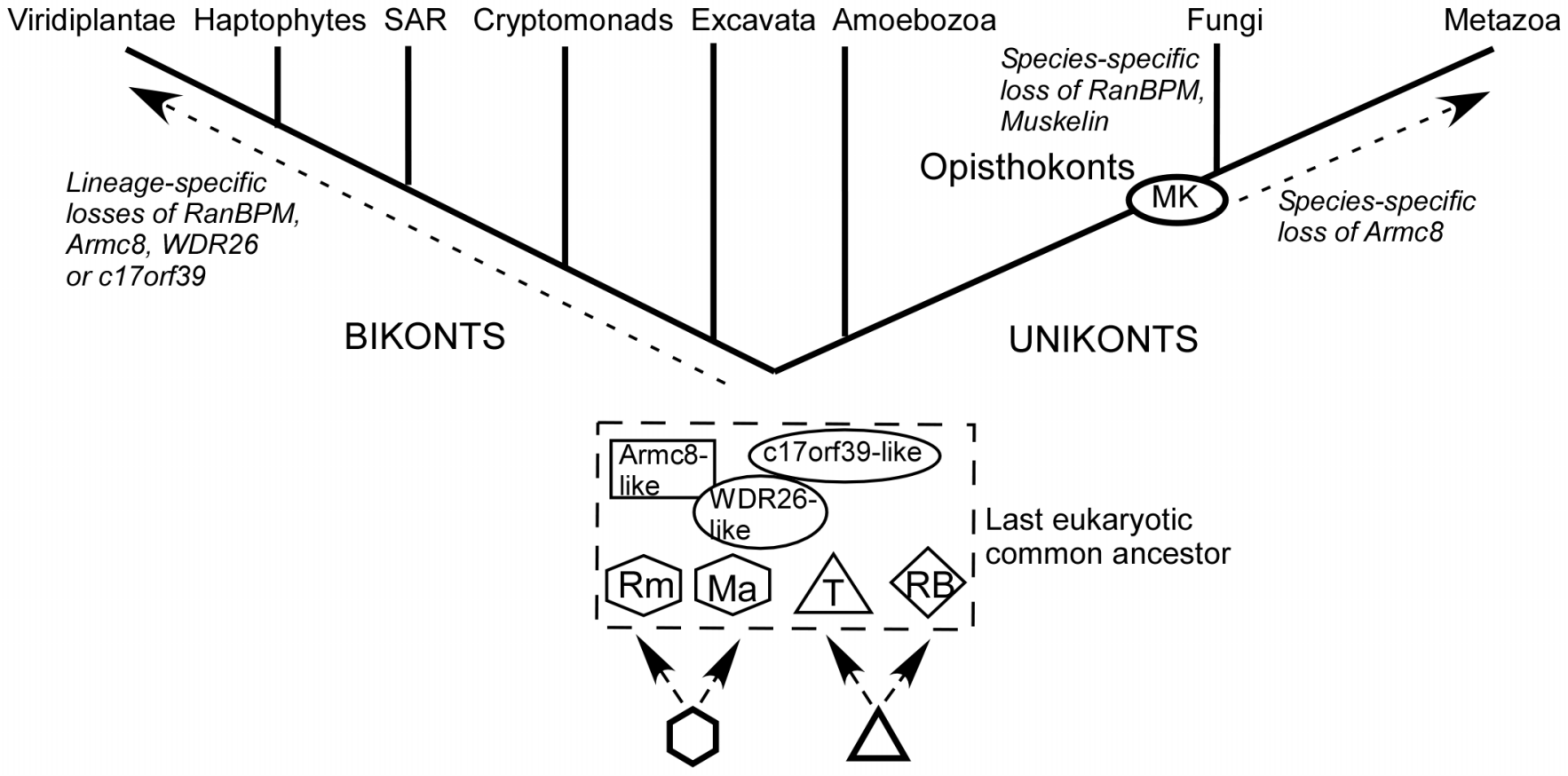
Model for the evolution of the MRCTLH complex. The model proposes that all proteins except muskelin were present in the last eukaryotic common ancestor, also that RanBPM and TWA1 vs MAEA and Rmnd5 share common ancestors, respectively. Rm = Rmnd5; Ma = MAEA; T = TWA1; RB = RanBPM. See Discussion for details.

Starting from extensive sequence database searches, we have combined molecular, phylogenetic, and initial experimental analyses to better understand the molecular phylogeny of the proteins of the MRCTLH/GID complex. Our data reveal that most components are conserved throughout multiple unikont and bikont eukaryotic super-groups. A consensus view of eukaryotic relationships includes six extant supergroups, yet the relationships between haptophytes, crytophytes and other plastid-carrying lineages remain contentious and debated [54]–[58]. Our findings lead us to propose the model that all complex components except muskelin were present in the last eukaryotic common ancestor (Fig. 7). No MRCTLH-like proteins were identified in prokaryotes. These findings can be integrated with previous publications on the biochemical composition of MRCTLH/GID complexes identified in mammalian cells, *S. cerevisiae* and *A. thaliana* cultured cells [9], [12], [14]. In relation to the eight components of the *S. cerevisiae* GID complex, the mammalian complex lacks c17orf39/GID4 and contains muskelin instead of WDR26/GID7 [9], [14]. The *A. thaliana* complex, albeit isolated as RanBPM-associated proteins, includes WDR26 but lacks Armc8/GID5 and c17orf39/GID4, even though both of these proteins are encoded in the *A. thaliana* genome [12]. The most consistent components of the biochemically-isolated complexes – TWA1, MAEA, Rmnd5, RanBPM – are also amongst the most conserved. We suggest that these components may form a “core complex”, with other components such as Armc8, c17orf39, muskelin or WDR26 having more peripheral roles to integrate complex activity with distinct signals or pathways in different eukaryotic lineages. This concept would be in agreement with a study of GID complex topology by co-immunoprecipitation methods [59].

The inclusion of muskelin, an opisthokont innovation, within the complex via its activity as a RanBP9 binding protein is likely to have conferred new activities or new regulatory possibilities on the complex. For example, the functional link between muskelin, RanBP9 and ECM-cytoskeletal adhesion pathways might be opisthokont- or metazoan-specific [16]. Muskelin is represented in many fungi including *S. pombe* but is not encoded in the *S. cerevisiae* genome [13], [48 and this study]. It is now considered that *S. cerevisiae* is a member of a derived lineage [60], thus muskelin gene loss is likely a secondary trait in *S. cerevisiae*. A related role in the *S. cerevisiae* GID complex is taken by GID7, the homologue of mammalian WDR26 [9]. Because WDR26 is present in the complex of RanBPM-associated proteins isolated from *A. thaliana* [12] and its phylogenetic distribution includes all supergroups (Fig. 2), we propose that WDR26 proteins might have GID7-like roles in other lineages as well. Metazoa and fungi include many species that encode both WDR26 and muskelin. It will be of future interest to establish experimentally whether replacement of WDR26 with muskelin is specific to the mammalian MRCTLH complex. The binding partner of muskelin, RanBPM, is represented in both unikont and bikont eukaryotes, yet RanBPM is also relatively highly variable in sequence, with regard to extensions of its N-terminal region and its LisH/CTLH domains. Multiple binding partners of the mammalian paralogues, RanBP9 and RanBP10, have been identified in yeast two hybrid screens [21]–[23], [61], [62], giving rise to the notion that RanBP9 could act as an adaptor that brings substrate proteins into proximity of the complex. The data suggest that RanBPM's interactions and function(s) are susceptible to rapid evolutionary change.

Our phylogenetic and sequence conservation analyses of the shared LisH and CTLH domains provided additional insight into the evolutionary relationships of RanBPM, TWA1, MAEA and Rmnd5. In general, LisH/CTLH domains have roles in mediating dimerisation [46], [49], and the presence of these domains in multiple proteins within the MRCTLH complex led initially to proposals of functional equivalence [14]. The phylogenetic analysis demonstrates that Rmnd5 and MAEA LisH/CTLH sequences form a more closely related pair, as do the TWA1 and RanBPM sequences (Fig. 2). These pairings were substantiated by the sequence Logos: the sequence Logos of TWA1 and RanBPM are each more similar to the overall LisH and CTLH consensus, whereas those of Rmnd5 and MAEA are similar to each other in containing well-conserved charged residues within the N-terminal α-helix (Fig. 3) or distinct residues (eg, tryptophan; Fig. 4), that are not present in the general consensus for these domains. The relationship between Rmnd5 and MAEA was clarified further by the identification of a conserved variant RING domain in MAEA as well as in Rmnd5 (Fig. 6). Based on these identifications, we propose that the encoding genes originated through gene duplications prior to the emergence of the last eukaryotic common ancestor (Fig. 7). The phylogenetic analysis of all sequences in the dataset revealed an unexpected relationship between the LisH and CTLH domains of muskelin and those of Rmnd5 and MAEA (Figure S3). The Sequence Logos clarified that the LisH/CTLH domains of all three proteins display conservation of multiple charged residues (Figs, 3, 4 and [16]). In view that no protein sequences representing “intermediates” between MAEA/Rmnd5 and muskelin were identified in our searches, and that the specific conserved residues in the sequence Logos of the muskelin domains are distinct from those of Rmnd5 and MAEA, we propose that the apparent phylogenetic relationship of the muskelin LisH and CTLH domains may represent an instance of convergent evolution.

The implication of distinct functional activities amongst the LisH and CTLH domains of RanBPM, TWA1, MAEA and Rmnd5 was strengthened by a comparison of the sub-cellular localisations of domain deletion proteins and the full-length proteins. To make this comparison, it was necessary to express tagged proteins in cells. The expressed full-length proteins had localisations equivalent to initial reports on the localisations of the endogenous proteins [12], [14]–[16]. Although the majority location of endogenous RanBP9 is in the cytoplasm, a nuclear pool has been identified by cell fractionation [16] or immunofluorescence [12]. The biochemical isolation procedures have utilised predominantly cytoplasmic extracts, thus it appears that components are available to interact in either the cytoplasm or nucleus. Our analysis revealed distinct results for the localisation of each protein when truncated. For RanBPM or MAEA the main effect of LisH deletion appeared to be on protein folding or stability. The localisation of TWA1 was not affected by deletion of its LisH domain, suggesting that this small protein diffuses through nuclear pores (Fig. 5). The major role of this domain in TWA1 may relate to oligomerisation. Indeed, TWA1 is necessary for assembly of the yeast complex [63]. In contrast, deletion of the LisH domain of Rmnd5a has a major effect on subcellular localisation. In view that Rmnd5a has a molecular mass of 44 kDa, that should permit diffusion through nuclear pores, this result suggests that the LisH domain of Rmnd5a might contain a nuclear localisation signal or interact preferentially with binding partners in the nucleus. A positively charged motif in the muskelin LisH domain functions as a nuclear localisation signal [16]. Beyond the scope of this study, development of domain chimeras or mutation of identified highly conserved residues will enable further exploration of these findings.

Sequence analysis of the C-terminal regions of Rmnd5 and MAEA demonstrated strong conservation of the RING domain of Rmnd5 and its similarity to the RING-HC and RING-S/T subgroups of RING-E3s. Indeed, mediation of E3 ligase activity has been demonstrated within the GID complex [9]. Human Rmnd5b has polyubiqutination activity in combination with multiple E2 enzymes [64] and *in vitro* E3 ligase activity has been reported for a Rmnd5 orthologue of *Lotus japonicus* [65]. Thus, E3 ligase activity of Rmnd5 appears widely conserved. A striking finding from the comparison of all MAEA and Rmnd5 protein sequences in our dataset was the identification that a RING-like domain is conserved at the C-terminus of MAEA. We speculate that the atypical strongly conserved prolines of the MAEA RING domain may assist to maintain the RING fold in the absence of zinc chelation. The atypical distribution of cysteine residues within the Maea RING domain does not immediately suggest an active E3 ligase. GID9 has been identified to act as an E3 ligase as a hetero-mer with GID2/Rmnd5 [66]. The identification that GID2/GID9 work together in E3 ligase activity is congruent with our molecular evolutionary data on the high conservation of MAEA and Rmnd5 and further supports the model of a common ancestry and related functions of these two proteins.

A well-characterised function of S. *cerevisiae* GID complex is to mediate the poly-ubiquitination of FBPase under glucose-rich conditions, leading to its degradation by the proteosome [9]. FBPase is a major enzyme of gluconeogenesis that catalyses the hydrolysis of fructose 1,6-bisphosphate to fructose 6-phosphate and inorganic phosphate. FBPase is widely represented in eukaryotes and archaebacteria [67], [68]. Eukaryotic and bacterial FBPases are strongly conserved and exhibit FBPase activity exclusively whereas bifunctional FBP aldolases/phosphatases are very well conserved in archaea and some bacterial species, indicating an early origin for gluconeogenesis compared to glycolysis [69]. Furthermore, the glucose catabolism pathways of archaea are significantly more different to those of bacteria and eukaryotes than the glucose anabolism pathways [70]. In considering whether FBPase ubiquitination might be an ancient and central activity of MRCTLH complex, we find that not all eukaryotes that encode MRCTLH complex components also encode FBPase. Prime examples are microsporidian fungi such as *Encephalitozoon cuniculi* and the excavate, *N. gruberi*, considered to be an organism that has retained features of the eukaryotic ancestor. *N. gruberi* encodes TWA1, MAEA, Rmnd5 and WDR26 and lacks FBPase [71]. In future, experimental studies in this species could be informative for identifying possible ancestral functions.

In conclusion, all MRCTLH proteins except muskelin appear to have been present in the last eukaryotic common ancestor. TWA1, Maea, Rmnd5 are the most highly conserved and RanBPM and WDR26 are also well-conserved across unikonts and bikonts. Armc8 and c17orf39 are well-conserved in unikonts but have been lost from many bikont lineages. These data add to growing evidence for the complexity of the eukaryotic ancestor. Based on phylogenetic and domain sequence conservation analyses we propose that TWA1 and RanBPM versus MAEA and Rmnd5 arose originally through gene duplications. Our data implicate conserved roles of both Rmnd5 and MAEA as E3 ubiquitin ligases within the MRCTLH complex, whereas RanBPM appears fast-evolving and Armc8 and c17orf39 appear dispensible, particularly in bikonts. TWA1 is highly conserved and might function as an oligomerising scaffold within the complex. These molecular phylogenetic insights provide a coherent framework for future experimental investigations of the MRCTLH complex in different lineages of eukaryotes.

## Supporting Information

Figure S1 Phylogenetic distribution of each of the eight MRCTLH proteins in eukaryotes.
(PDF)

Figure S2 The phylogenetic relationships of RanBPM, TWA1, MAEA and Rmnd5, based on their LisH/CTLH regions. 922 sequences spanning the LisH and CTLH regions were aligned as described in the Methods. The Newick output is presented here as a circular tree with proportionate branch lengths, with every entry identified by its GenBank accession number. Key: background shading denotes protein identities: blue = RanBPM, grey = TWA1, yellow = Rmnd5, pink = muskelin and green = MAEA. Colours of branches, text and outer band denote the taxonomical grouping of each species. Dark red = choanoflagellates, red = metazoa, blue = fungi, green = plants, brown = SAR, purple = amoebozoa, pink = excavate, black = independent opisthokont, orange = cryptophyte and dark grey = haptophyte, respectively. clustered by either algorithm; scale bar indicates substitutions/site. Branch support values are bootstrap values based on bootstop autoconvergence at 650 cycles.
(PDF)

Figure S3 Expression of tagged MAEA, TWA1, Rmnd5a and their N- or C-terminal domain deletions. Proteins were transiently expressed by plasmid transfection into COS-7 cells as described in the Methods. After 48 h, whole cell lysates were prepared in SDS-PAGE sample buffer, resolved on 12.5% polyacrylamide gels under reducing conditions and transferred to PVDF membranes for immunoblotting with antibodies to the tags as indicated in the figure panels. All proteins had the expected apparent molecular masses. Protein levels of Rmnd5a were markedly lower than those of MAEA when compared on the same blot (middle panel). Immunoblot analysis of RanBP9 domains was published in [16]. FL = full-length; N = N-terminal domains, C = C-terminal domains as in Fig. 5. Molecular mass markers are given in kDa.
(PDF)

Table S1 Oligonucleotides used for preparation of expression constructs.
(PDF)
