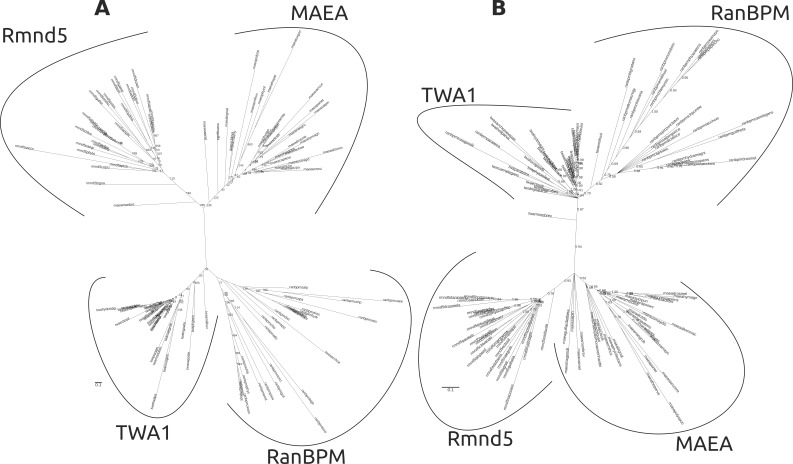# Correction: Molecular Phylogeny of a RING E3 Ubiquitin Ligase, Conserved in Eukaryotic Cells and Dominated by Homologous Components, the Muskelin/RanBPM/CTLH Complex

**DOI:** 10.1371/annotation/1e464689-3c86-4399-b229-1e00d65593a5

**Published:** 2013-11-08

**Authors:** Ore Francis, Fujun Han, Josephine C. Adams

Figure 2 was published as a duplicate of Figure 4. Please find the correct Figure 2 here: 

**Figure:**