# Supplementary material for: Molecular Phylogeny of a RING E3 Ubiquitin Ligase, Conserved in Eukaryotic Cells and Dominated by Homologous Components, the Muskelin/RanBPM/CTLH Complex
Source: PLoS One. 2013 Oct 15;8(10):e75217. doi: 10.1371/journal.pone.0075217 (PMC3797097; doi:10.1371/journal.pone.0075217)
Supplement: Figure S1 — Phylogenetic distribution of each of the eight MRCTLH proteins in eukaryotes. (PDF) [file pone.0075217.s001.pdf]

Figure S1

The taxonomical trees were generated in PHYLIP format with the NCBI XML tree tool (http://ncbi.nlm.nih.gov/tools/cobalt/cobalt.cgi). PHYLIP files were concatenated and rendered using Treedyn. Each taxonomic lineage is colour-coded. Accession numbers for the sequence of each species (GenBank gi and/or Uniprot) are given on the right-hand side of each tree.

Tree1. Taxonomic tree of the phylogenetic distribution of muskelin in eukaryotes.
Tree2. Taxonomic tree of the phylogenetic distribution of Rmnd5 in eukaryotes.
Tree3. Taxonomic tree of the phylogenetic distribution of MAEA in eukaryotes.
Tree4. Taxonomic tree of the phylogenetic distribution of TWA1 in eukaryotes.
Tree5. Taxonomic tree of the phylogenetic distribution of RanBPM in eukaryotes.
Tree 6. Taxonomic tree of the phylogenetic distribution of WDR26 in eukaryotes.
Tree 7. Taxonomic tree of the phylogenetic distribution of Armc8 in eukaryotes.
Tree 8. Taxonomic tree of the phylogenetic distribution of c17orf39 in eukaryotes.

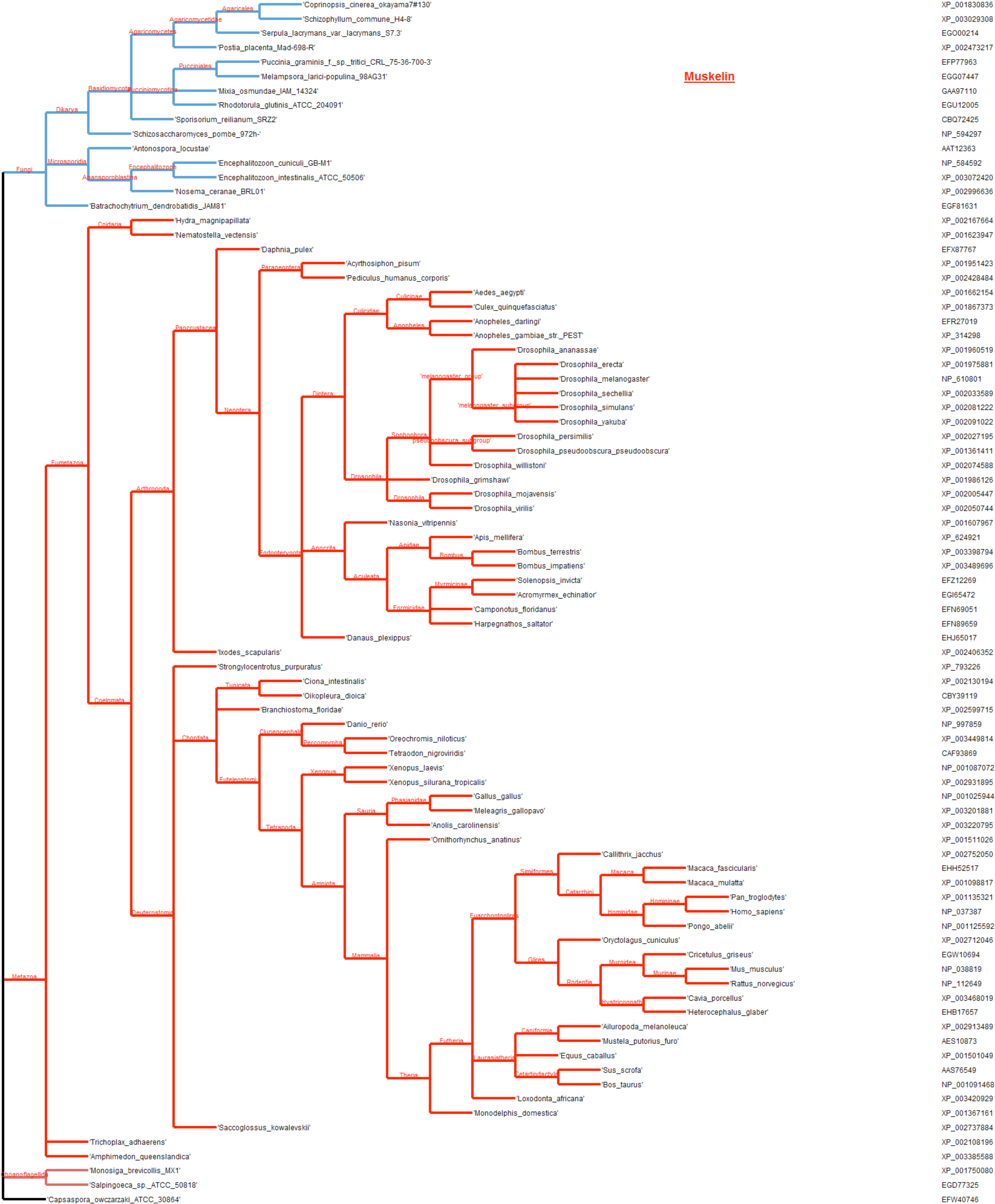
Muskelin
'Coprinopsis_cinerea_okayama7#130' XP_001830836
'Schizophyllum_commune_H4-8' XP_003029308
'Serpula_lacrymans_var._lacrymans_S7.3' EGO00214
'Postia_placenta_Mad-698-R' XP_002473217
'Puccinia_graminis_f._sp._tritici_CRL_75-36-700-3' EFP77963
'Melampsora_larici-populina_98AG31' EGG07447
'Mixia_osmundae_IAM_14324' GAA97110
'Rhodotorula_glutinis_ATCC_204091' EGU12005
'Sporisorium_reilianum_SRZ2' CBQ72425
'Schizosaccharomyces_pombe_972h-' NP_594297
'Antonospora_locustae' AAT12363
'Encephalitozoon_cuniculi_GB-M1' NP_584592
'Encephalitozoon_intestinalis_ATCC_50506' XP_003072420
'Nosema_ceranae_BRL01' XP_002996636
'Batrachochytrium_dendrobatidis_JAM81' EGF81631
'Hydra_magnipapillata' XP_002167664
'Nematostella_vectensis' XP_001623947
'Daphnia_pulex' EFX87767
'Acyrthosiphon_pisum' XP_001951423
'Pediculus_humanus_corporis' XP_002428484
'Aedes_aegypti' XP_001662154
'Culex_quinquefasciatus' XP_001867373
'Anopheles_darlingi' EFR27019
'Anopheles_gambiae_str._PEST' XP_314298
'Drosophila_ananassae' XP_001960519
'Drosophila_erecta' XP_001975881
'Drosophila_melanogaster' NP_610801
'Drosophila_sechellia' XP_002033589
'Drosophila_simulans' XP_002081222
'Drosophila_yakuba' XP_002091022
'Drosophila_persimilis' XP_002027195
'Drosophila_pseudoobscura_pseudoobscura' XP_001361411
'Drosophila_willistoni' XP_002074588
'Drosophila_grimshawi' XP_001986126
'Drosophila_mojavensis' XP_002005447
'Drosophila_virilis' XP_002050744
'Nasonia_vitripennis' XP_001607967
'Apis_mellifera' XP_624921
'Bombus_terrestris' XP_003398794
'Bombus_impatiens' XP_003489696
'Solenopsis_invicta' EFZ12269
'Acromyrmex_echinatior' EGI65472
'Camponotus_floridanus' EFN69051
'Harpegnathos_saltator' EFN89659
'Danaus_plexippus' EHJ65017
'Ixodes_scapularis' XP_002406352
'Strongylocentrotus_purpuratus' XP_793226
'Ciona_intestinalis' XP_002130194
'Oikopleura_dioica' CBY39119
'Branchiostoma_floridae' XP_002599715
'Danio_rerio' NP_997859
'Oreochromis_niloticus' XP_003449814
'Tetraodon_nigroviridis' CAF93869
'Xenopus_laevis' NP_001087072
'Xenopus_silurana_tropicalis' XP_002931895
'Gallus_gallus' NP_001025944
'Meleagris_gallopavo' XP_003201881
'Anolis_carolinensis' XP_003220795
'Ornithorhynchus_anatinus' XP_001511026
'Callithrix_jacchus' XP_002752050
'Macaca_fascicularis' EHH52517
'Macaca_mulatta' XP_001098817
'Pan_troglodytes' XP_001135321
'Homo_sapiens' NP_037387
'Pongo_abelii' NP_001125592
'Oryctolagus_cuniculus' XP_002712046
'Cricetulus_griseus' EGW10694
'Mus_musculus' NP_038819
'Rattus_norvegicus' NP_112649
'Cavia_porcellus' XP_003468019
'Heterocephalus_glaber' EHB17657
'Ailuropoda_melanoleuca' XP_002913489
'Mustela_putorius_furo' AES10873
'Equus_caballus' XP_001501049
'Sus_scrofa' AAS76549
'Bos_taurus' NP_001091468
'Loxodonta_africana' XP_003420929
'Monodelphis_domestica' XP_001367161
'Saccoglossus_kowalevskii' XP_002737884
'Trichoplax_adhaerens' XP_002108196
'Amphimedon_queenslandica' XP_003385588
'Monosiga_brevicollis_MX1' XP_001750080
'Salpingoeca_sp._ATCC_50818' EGD77325
'Capsaspora_owczarzaki_ATCC_30864' EFW40746

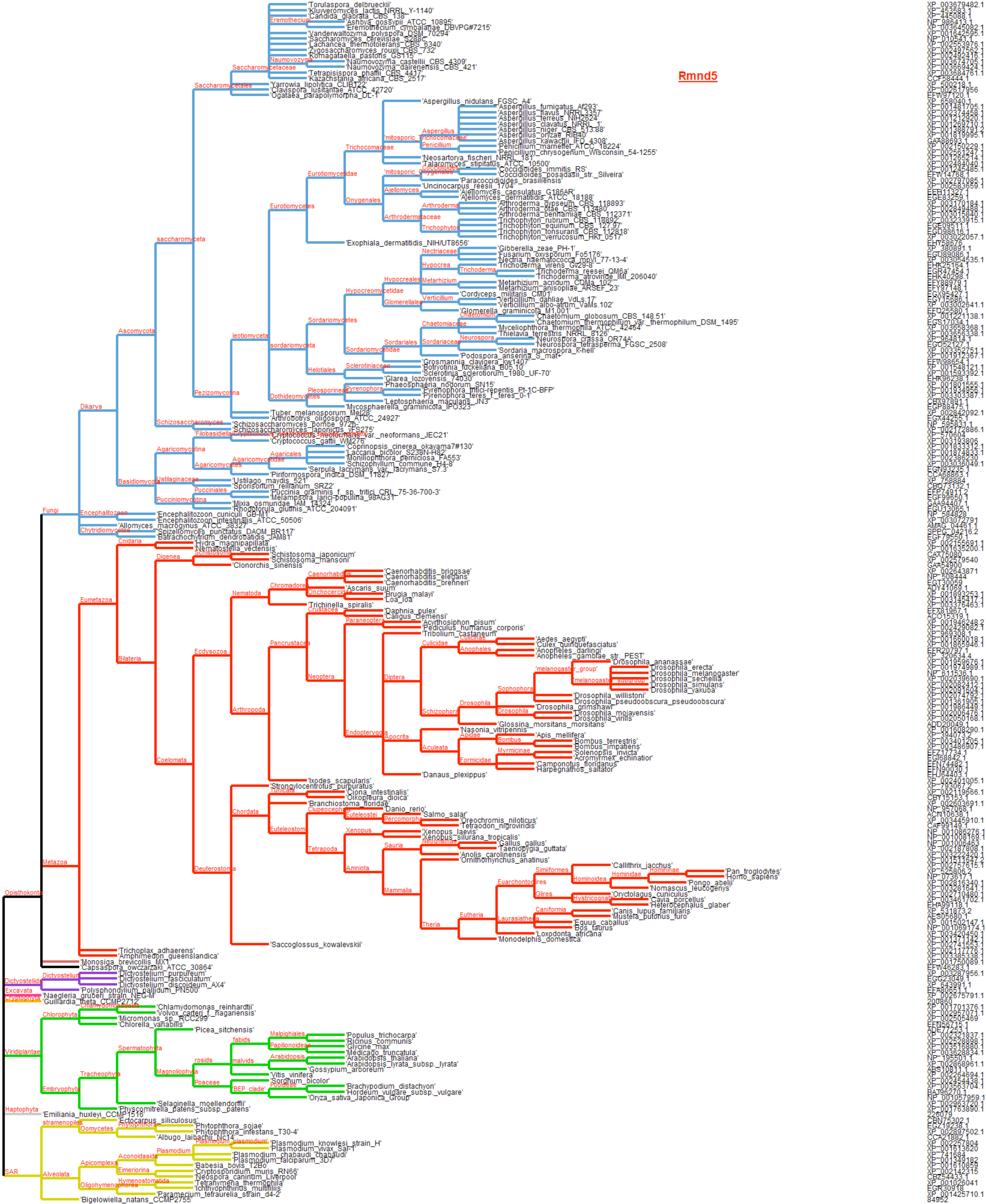

Rmnd5
'Torulaspora_delbrueckii'
'Kluyveromyces_lactis_NRRL_Y-1140'
'Candida_glabrata_CBS_138'
'Ashbya_gossypii_ATCC_10895'
'Eremothecium_cymbalariae_DBVPG#7215'
'Vanderwaltozyma_polyspora_DSM_70294'
'Saccharomyces_cerevisiae_S288c'
'Lachancea_thermotolerans_CBS_6340'
'Zygosaccharomyces_rouxii_CBS_732'
'Komagataella_pastoris_GS115'
'Naumovozyma_castellii_CBS_4309'
'Naumovozyma_dairenensis_CBS_421'
'Tetrapisispora_phaffii_CBS_4417'
'Kazachstania_africana_CBS_2517'
'Yarrowia_lipolytica_CLIB122'
'Clavispora_lusitaniae_ATCC_42720'
'Ogataea_parapolymorpha_DL-1'
'Aspergillus_nidulans_FGSC_A4'
'Aspergillus_fumigatus_Af293'
'Aspergillus_flavus_NRRL3357'
'Aspergillus_terreus_NIH2624'
'Aspergillus_clavatus_NRRL_1'
'Aspergillus_niger_CBS_513.88'
'Aspergillus_oryzae_RIB40'
'Aspergillus_kawachii_IFO_4308'
'Penicillium_marneffei_ATCC_18224'
'Penicillium_chrysogenum_Wisconsin_54-1255'
'Neosartorya_fischeri_NRRL_181'
'Talaromyces_stipitatus_ATCC_10500'
'Coccidioides_immitis_RS'
'Coccidioides_posadasii_str._Silveira'
'Paracoccidioides_brasiliensis'
'Uncinocarpus_reesii_1704'
'Ajellomyces_capsulatus_G186AR'
'Ajellomyces_dermatitidis_ATCC_18188'
'Arthroderma_gypseum_CBS_118893'
'Arthroderma_otae_CBS_113480'
'Arthroderma_benhamiae_CBS_112371'
'Trichophyton_rubrum_CBS_118892'
'Trichophyton_equinum_CBS_127.97'
'Trichophyton_tonsurans_CBS_112818'
'Trichophyton_verrucosum_HKI_0517'
'Exophiala_dermatitidis_NIH/UT8656'
'Gibberella_zeae_PH-1'
'Fusarium_oxysporum_Fo5176'
'Nectria_haematococca_mpVI_77-13-4'
'Trichoderma_virens_Gv29-8'
'Trichoderma_reesei_QM6a'
'Trichoderma_atroviride_IMI_206040'
'Metarhizium_acridum_CQMa_102'
'Metarhizium_anisopliae_ARSEF_23'
'Cordyceps_militaris_CM01'
'Verticillium_dahliae_VdLs.17'
'Verticillium_albo-atrum_VaMs.102'
'Glomerella_graminicola_M1.001'
'Chaetomium_globosum_CBS_148.51'
'Chaetomium_thermophilum_var._thermophilum_DSM_1495'
'Myceliophthora_thermophila_ATCC_42464'
'Thielavia_terrestris_NRRL_8126'
'Neurospora_crassa_OR74A'
'Neurospora_tetrasperma_FGSC_2508'
'Sordaria_macrospora_k-hell'
'Podospora_anserina_S_mat+'
'Grosmannia_clavigera_kw1407'
'Botryotinia_fuckeliana_B05.10'
'Sclerotinia_sclerotiorum_1980_UF-70'
'Glarea_lozoyensis_74030'
'Phaeosphaeria_nodorum_SN15'
'Pyrenophora_tritici-repentis_Pt-1C-BFP'
'Pyrenophora_teres_f._teres_0-1'
'Leptosphaeria_maculans_JN3'
'Mycosphaerella_graminicola_IPO323'
'Tuber_melanosporum_Mel28'
'Arthrobotrys_oligospora_ATCC_24927'
'Schizosaccharomyces_pombe_972h-'
'Schizosaccharomyces_japonicus_yFS275'
'Cryptococcus_neoformans_var._neoformans_JEC21'
'Cryptococcus_gattii_WM276'
'Coprinopsis_cinerea_okayama7#130'
'Laccaria_bicolor_S238N-H82'
'Moniliophthora_perniciosa_FA553'
'Schizophyllum_commune_H4-8'
'Serpula_lacrymans_var._lacrymans_S7.3'
'Piriformospora_indica_DSM_11827'
'Ustilago_maydis_521'
'Sporisorium_reilianum_SRZ2'
'Puccinia_graminis_f._sp._tritici_CRL_75-36-700-3'
'Melampsora_larici-populina_98AG31'
'Mixia_osmundae_IAM_14324'
'Rhodotorula_glutinis_ATCC_204091'
'Encephalitozoon_cuniculi_GB-M1'
'Encephalitozoon_intestinalis_ATCC_50506'
'Allomyces_macrogynus_ATCC_38327'
'Spizellomyces_punctatus_DAOM_BR117'
'Batrachochytrium_dendrobatidis_JAM81'
'Hydra_magnipapillata'
'Nematostella_vectensis'
'Schistosoma_japonicum'
'Schistosoma_mansoni'
'Clonorchis_sinensis'
'Caenorhabditis_briggsae'
'Caenorhabditis_elegans'
'Caenorhabditis_brenneri'
'Ascaris_suum'
'Brugia_malayi'
'Loa_loa'
'Trichinella_spiralis'
'Daphnia_pulex'
'Caligus_clemensi'
'Acyrthosiphon_pisum'
'Pediculus_humanus_corporis'
'Tribolium_castaneum'
'Aedes_aegypti'
'Culex_quinquefasciatus'
'Anopheles_darlingi'
'Anopheles_gambiae_str._PEST'
'Drosophila_ananassae'
'Drosophila_erecta'
'Drosophila_melanogaster'
'Drosophila_sechellia'
'Drosophila_simulans'
'Drosophila_yakuba'
'Drosophila_willistoni'
'Drosophila_pseudoobscura_pseudoobscura'
'Drosophila_grimshawi'
'Drosophila_mojavensis'
'Drosophila_virilis'
'Glossina_morsitans_morsitans'
'Nasonia_vitripennis'
'Apis_mellifera'
'Bombus_terrestris'
'Bombus_impatiens'
'Solenopsis_invicta'
'Acromyrmex_echinatior'
'Camponotus_floridanus'
'Harpegnathos_saltator'
'Danaus_plexippus'
'Ixodes_scapularis'
'Strongylocentrotus_purpuratus'
'Ciona_intestinalis'
'Oikopleura_dioica'
'Branchiostoma_floridae'
'Danio_rerio'
'Salmo_salar'
'Oreochromis_niloticus'
'Tetraodon_nigroviridis'
'Xenopus_laevis'
'Xenopus_silurana_tropicalis'
'Gallus_gallus'
'Taeniopygia_guttata'
'Anolis_carolinensis'
'Ornithorhynchus_anatinus'
'Callithrix_jacchus'
'Pan_troglodytes'
'Homo_sapiens'
'Pongo_abelii'
'Nomascus_leucogenys'
'Oryctolagus_cuniculus'
'Cavia_porcellus'
'Heterocephalus_glaber'
'Canis_lupus_familiaris'
'Mustela_putorius_furo'
'Equus_caballus'
'Bos_taurus'
'Loxodonta_africana'
'Monodelphis_domestica'
'Saccoglossus_kowalevskii'
'Trichoplax_adhaerens'
'Amphimedon_queenslandica'
'Monosiga_brevicollis_MX1'
'Capsaspora_owczarzaki_ATCC_30864'
'Dictyostelium_purpureum'
'Dictyostelium_fasciculatum'
'Dictyostelium_discoideum_AX4'
'Polysphondylium_pallidum_PN500'
'Naegleria_gruberi_strain_NEG-M'
'Guillardia_theta_CCMP2712'
'Chlamydomonas_reinhardtii'
'Volvox_carteri_f._nagariensis'
'Micromonas_sp._RCC299'
'Chlorella_variabilis'
'Picea_sitchensis'
'Populus_trichocarpa'
'Ricinus_communis'
'Glycine_max'
'Medicago_truncatula'
'Arabidopsis_thaliana'
'Arabidopsis_lyrata_subsp._lyrata'
'Gossypium_arboreum'
'Vitis_vinifera'
'Sorghum_bicolor'
'Brachypodium_distachyon'
'Hordeum_vulgare_subsp._vulgare'
'Oryza_sativa_Japonica_Group'
'Selaginella_moellendorffii'
'Physcomitrella_patens_subsp._patens'
'Emiliania_huxleyi_CCMP1516'
'Ectocarpus_siliculosus'
'Phytophthora_sojae'
'Phytophthora_infestans_T30-4'
'Albugo_laibachii_Nc14'
'Plasmodium_knowlesi_strain_H'
'Plasmodium_vivax_Sal-1'
'Plasmodium_chabaudi_chabaudi'
'Plasmodium_falciparum_3D7'
'Babesia_bovis_T2Bo'
'Cryptosporidium_muris_RN66'
'Neospora_caninum_Liverpool'
'Tetrahymena_thermophila'
'Ichthyophthirius_multifiliis'
'Paramecium_tetraurelia_strain_d4-2'
'Bigelowiella_natans_CCMP2755'
Opisthokonta
Fungi
Dikarya
Ascomycota
saccharomyceta
Saccharomycetales
Saccharomycetaceae
Eremothecium
Naumovozyma
Pezizomycotina
leotiomyceta
Eurotiomycetes
Eurotiomycetidae
Trichocomaceae
Aspergillus
Penicillium
Onygenales
Ajellomyces
Arthrodermataceae
Arthroderma
Trichophyton
sordariomyceta
Sordariomycetes
Hypocreomycetidae
Hypocreales
Nectriaceae
Hypocrea
Trichoderma
Metarhizium
Glomerellales
Verticillium
Sordariomycetidae
Sordariales
Chaetomiaceae
Sordariaceae
Neurospora
Leotiomycetes
Helotiales
Sclerotiniaceae
Dothideomycetes
Pleosporineae
Pyrenophora
Schizosaccharomyces
Basidiomycota
Agaricomycotina
Agaricomycetes
Agaricomycetidae
Agaricales
Ustilaginaceae
Pucciniomycotina
Pucciniales
Encephalitozoon
Chytridiomycetes
Metazoa
Eumetazoa
Cnidaria
Bilateria
Digenea
Schistosoma
Coelomata
Ecdysozoa
Nematoda
Chromadorea
Caenorhabditis
Onchocercidae
Arthropoda
Pancrustacea
Crustacea
Neoptera
Paraneoptera
Endopterygota
Diptera
Culicidae
Culicinae
Anopheles
Schizophora
Drosophila
Sophophora
'melanogaster_group'
'melanogaster_subgroup'
Apocrita
Aculeata
Apidae
Bombus
Formicidae
Myrmicinae
Deuterostomia
Chordata
Tunicata
Euteleostomi
Clupeocephala
Euteleostei
Percomorpha
Xenopus
Tetrapoda
Amniota
Sauria
Neognathae
Mammalia
Theria
Eutheria
Euarchontoglires
Simiiformes
Hominoidea
Hominidae
Homininae
Glires
Hystricognathi
Laurasiatheria
Caniformia
Dictyosteliida
Dictyostelium
Excavata
Cryptophyta
Viridiplantae
Chlorophyta
Chlamydomonadales
Embryophyta
Tracheophyta
Spermatophyta
Magnoliophyta
rosids
fabids
Malpighiales
Papilionoideae
malvids
Arabidopsis
Poaceae
BEP_clade
Pooideae
Haptophyta
SAR
stramenopiles
Oomycetes
Phytophthora
Alveolata
Apicomplexa
Aconoidasida
Plasmodium
Eimeriorina
Oligohymenophorea
Hymenostomatida

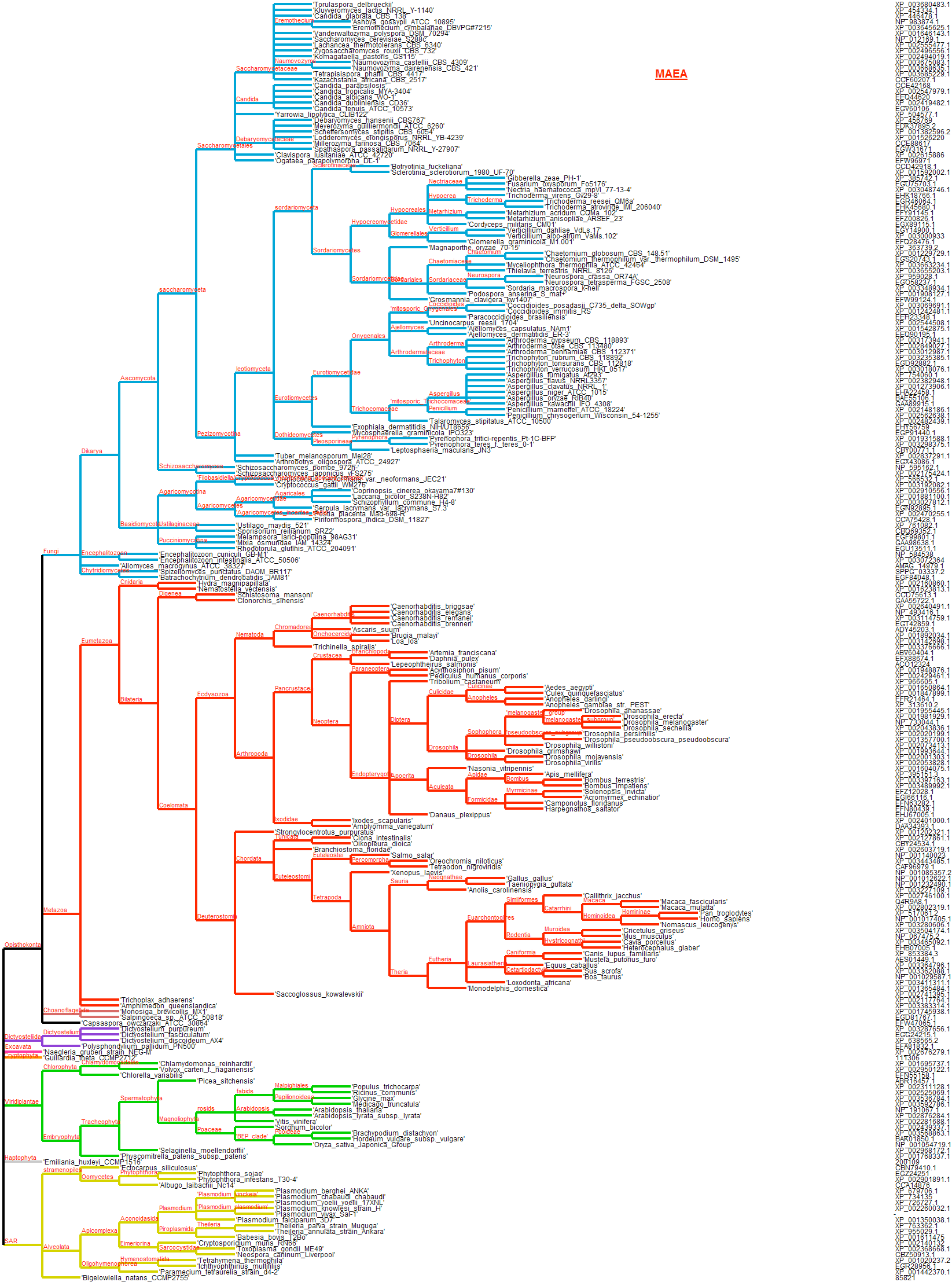
MAEA

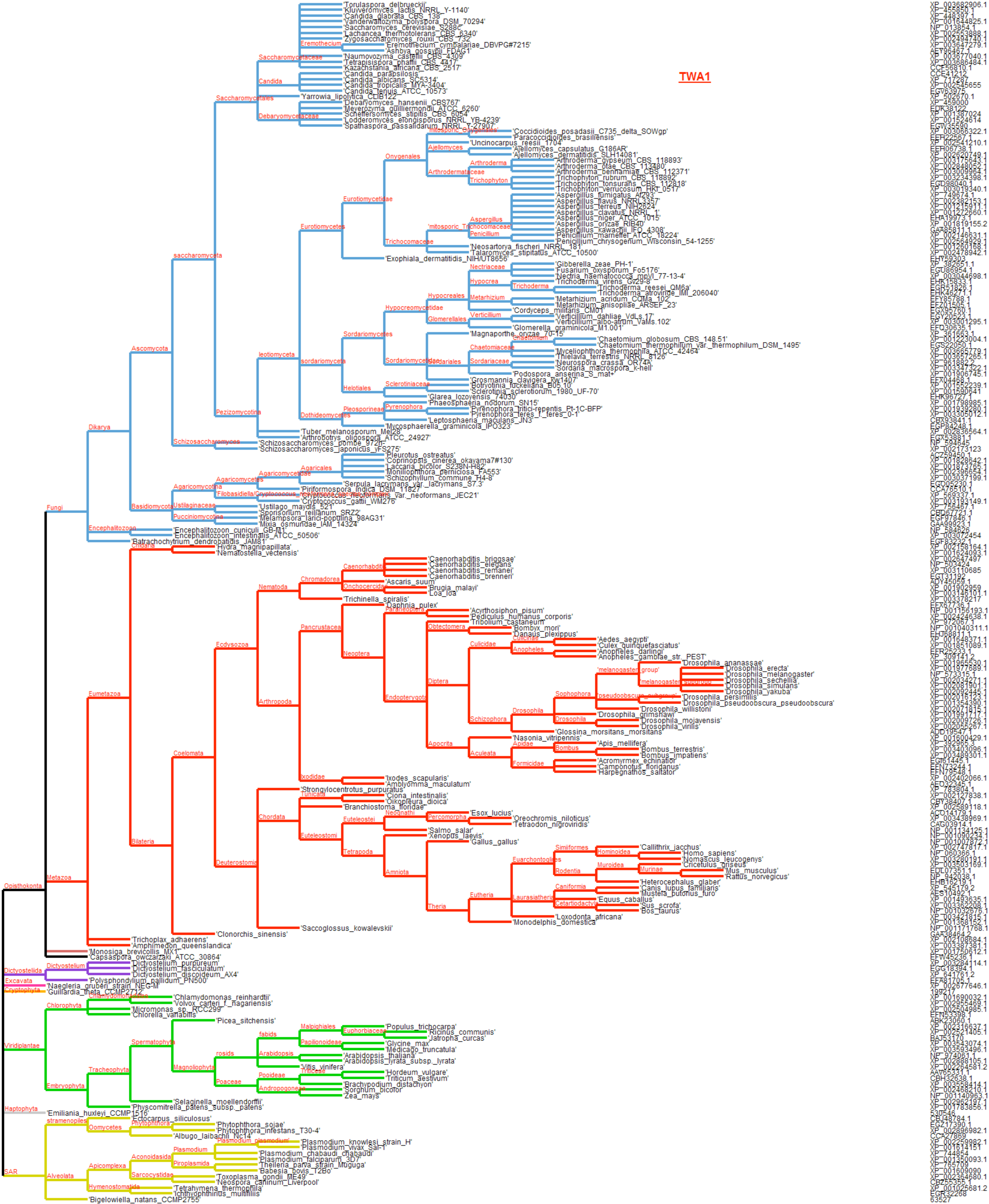
TWA1
Opisthokonta
Fungi
Dikarya
Ascomycota
saccharomyceta
Saccharomycetales
Saccharomycetaceae
Eremothecium
Candida
Debaryomycetaceae
'Torulaspora_delbrueckii'
'Kluyveromyces_lactis_NRRL_Y-1140'
'Candida_glabrata_CBS_138'
'Vanderwaltozyma_polyspora_DSM_70294'
'Saccharomyces_cerevisiae_S288c'
'Lachancea_thermotolerans_CBS_6340'
'Zygosaccharomyces_rouxii_CBS_732'
'Eremothecium_cymbalariae_DBVPG#7215'
'Ashbya_gossypii_FDAG1'
'Naumovozyma_castellii_CBS_4309'
'Tetrapisispora_phaffii_CBS_4417'
'Kazachstania_africana_CBS_2517'
'Candida_parapsilosis'
'Candida_albicans_SC5314'
'Candida_tropicalis_MYA-3404'
'Candida_tenuis_ATCC_10573'
'Yarrowia_lipolytica_CLIB122'
'Debaryomyces_hansenii_CBS767'
'Meyerozyma_guilliermondii_ATCC_6260'
'Scheffersomyces_stipitis_CBS_6054'
'Lodderomyces_elongisporus_NRRL_YB-4239'
'Spathaspora_passalidarum_NRRL_Y-27907'
Pezizomycotina
leotiomyceta
Eurotiomycetes
Eurotiomycetidae
Onygenales
'mitosporic_Onygenales'
Ajellomyces
Arthrodermataceae
Arthroderma
Trichophyton
'Coccidioides_posadasii_C735_delta_SOWgp'
'Paracoccidioides_brasiliensis'
'Uncinocarpus_reesii_1704'
'Ajellomyces_capsulatus_G186AR'
'Ajellomyces_dermatitidis_SLH14081'
'Arthroderma_gypseum_CBS_118893'
'Arthroderma_otae_CBS_113480'
'Arthroderma_benhamiae_CBS_112371'
'Trichophyton_rubrum_CBS_118892'
'Trichophyton_tonsurans_CBS_112818'
'Trichophyton_verrucosum_HKI_0517'
Trichocomaceae
'mitosporic_Trichocomaceae'
Aspergillus
Penicillium
'Aspergillus_fumigatus_Af293'
'Aspergillus_flavus_NRRL3357'
'Aspergillus_terreus_NIH2624'
'Aspergillus_clavatus_NRRL_1'
'Aspergillus_niger_ATCC_1015'
'Aspergillus_oryzae_RIB40'
'Aspergillus_kawachii_IFO_4308'
'Penicillium_marneffei_ATCC_18224'
'Penicillium_chrysogenum_Wisconsin_54-1255'
'Neosartorya_fischeri_NRRL_181'
'Talaromyces_stipitatus_ATCC_10500'
'Exophiala_dermatitidis_NIH/UT8656'
sordariomyceta
Sordariomycetes
Hypocreomycetidae
Hypocreales
Nectriaceae
Hypocrea
Trichoderma
Metarhizium
Glomerellales
Verticillium
'Gibberella_zeae_PH-1'
'Fusarium_oxysporum_Fo5176'
'Nectria_haematococca_mpVI_77-13-4'
'Trichoderma_virens_Gv29-8'
'Trichoderma_reesei_QM6a'
'Trichoderma_atroviride_IMI_206040'
'Metarhizium_acridum_CQMa_102'
'Metarhizium_anisopliae_ARSEF_23'
'Cordyceps_militaris_CM01'
'Verticillium_dahliae_VdLs.17'
'Verticillium_albo-atrum_VaMs.102'
'Glomerella_graminicola_M1.001'
'Magnaporthe_oryzae_70-15'
Sordariomycetidae
Sordariales
Chaetomiaceae
Chaetomium
Sordariaceae
'Chaetomium_globosum_CBS_148.51'
'Chaetomium_thermophilum_var._thermophilum_DSM_1495'
'Myceliophthora_thermophila_ATCC_42464'
'Thielavia_terrestris_NRRL_8126'
'Neurospora_crassa_OR74A'
'Sordaria_macrospora_k-hell'
'Podospora_anserina_S_mat+'
Helotiales
Sclerotiniaceae
'Grosmannia_clavigera_kw1407'
'Botryotinia_fuckeliana_B05.10'
'Sclerotinia_sclerotiorum_1980_UF-70'
'Glarea_lozoyensis_74030'
Dothideomycetes
Pleosporineae
Pyrenophora
'Phaeosphaeria_nodorum_SN15'
'Pyrenophora_tritici-repentis_Pt-1C-BFP'
'Pyrenophora_teres_f._teres_0-1'
'Leptosphaeria_maculans_JN3'
'Mycosphaerella_graminicola_IPO323'
'Tuber_melanosporum_Mel28'
'Arthrobotrys_oligospora_ATCC_24927'
Schizosaccharomyces
'Schizosaccharomyces_pombe_972h-'
'Schizosaccharomyces_japonicus_yFS275'
Basidiomycota
Agaricomycotina
Agaricomycetes
Agaricomycetidae
Agaricales
'Pleurotus_ostreatus'
'Coprinopsis_cinerea_okayama7#130'
'Laccaria_bicolor_S238N-H82'
'Moniliophthora_perniciosa_FA553'
'Schizophyllum_commune_H4-8'
'Serpula_lacrymans_var._lacrymans_S7.3'
'Piriformospora_indica_DSM_11827'
Filobasidiella/Cryptococcus_neoformans_species_complex
'Cryptococcus_neoformans_var._neoformans_JEC21'
'Cryptococcus_gattii_WM276'
Ustilaginaceae
'Ustilago_maydis_521'
'Sporisorium_reilianum_SRZ2'
Pucciniomycotina
'Melampsora_larici-populina_98AG31'
'Mixia_osmundae_IAM_14324'
Encephalitozoon
'Encephalitozoon_cuniculi_GB-M1'
'Encephalitozoon_intestinalis_ATCC_50506'
'Batrachochytrium_dendrobatidis_JAM81'
Metazoa
Eumetazoa
Cnidaria
'Hydra_magnipapillata'
'Nematostella_vectensis'
Bilateria
Coelomata
Ecdysozoa
Nematoda
Chromadorea
Caenorhabditis
Onchocercidae
'Caenorhabditis_briggsae'
'Caenorhabditis_elegans'
'Caenorhabditis_remanei'
'Caenorhabditis_brenneri'
'Ascaris_suum'
'Brugia_malayi'
'Loa_loa'
'Trichinella_spiralis'
Arthropoda
Pancrustacea
'Daphnia_pulex'
Neoptera
Paraneoptera
Endopterygota
Obtectomera
'Acyrthosiphon_pisum'
'Pediculus_humanus_corporis'
'Tribolium_castaneum'
'Bombyx_mori'
'Danaus_plexippus'
Diptera
Culicidae
Culicinae
Anopheles
'Aedes_aegypti'
'Culex_quinquefasciatus'
'Anopheles_darlingi'
'Anopheles_gambiae_str._PEST'
Schizophora
Sophophora
Drosophila
'melanogaster_group'
'melanogaster_subgroup'
'pseudoobscura_subgroup'
'Drosophila_ananassae'
'Drosophila_erecta'
'Drosophila_melanogaster'
'Drosophila_sechellia'
'Drosophila_simulans'
'Drosophila_yakuba'
'Drosophila_persimilis'
'Drosophila_pseudoobscura_pseudoobscura'
'Drosophila_willistoni'
'Drosophila_grimshawi'
'Drosophila_mojavensis'
'Drosophila_virilis'
'Glossina_morsitans_morsitans'
Apocrita
'Nasonia_vitripennis'
Aculeata
Apidae
Bombus
Formicidae
'Apis_mellifera'
'Bombus_terrestris'
'Bombus_impatiens'
'Acromyrmex_echinatior'
'Camponotus_floridanus'
'Harpegnathos_saltator'
Ixodidae
'Ixodes_scapularis'
'Amblyomma_maculatum'
Deuterostomia
Chordata
'Strongylocentrotus_purpuratus'
Tunicata
'Ciona_intestinalis'
'Oikopleura_dioica'
'Branchiostoma_floridae'
Euteleostomi
Euteleostei
Neognathi
Percomorpha
'Esox_lucius'
'Oreochromis_niloticus'
'Tetraodon_nigroviridis'
'Salmo_salar'
Tetrapoda
'Xenopus_laevis'
Amniota
'Gallus_gallus'
Theria
Eutheria
Euarchontoglires
Simiiformes
Hominoidea
'Callithrix_jacchus'
'Homo_sapiens'
'Nomascus_leucogenys'
Rodentia
Muroidea
Murinae
'Cricetulus_griseus'
'Mus_musculus'
'Rattus_norvegicus'
'Heterocephalus_glaber'
Laurasiatheria
Caniformia
Cetartiodactyla
'Canis_lupus_familiaris'
'Mustela_putorius_furo'
'Equus_caballus'
'Sus_scrofa'
'Bos_taurus'
'Loxodonta_africana'
'Monodelphis_domestica'
'Saccoglossus_kowalevskii'
'Clonorchis_sinensis'
'Trichoplax_adhaerens'
'Amphimedon_queenslandica'
'Monosiga_brevicollis_MX1'
'Capsaspora_owczarzaki_ATCC_30864'
Dictyostelida
Dictyostelium
'Dictyostelium_purpureum'
'Dictyostelium_fasciculatum'
'Dictyostelium_discoideum_AX4'
'Polysphondylium_pallidum_PN500'
Excavata
'Naegleria_gruberi_strain_NEG-M'
Cryptophyta
'Guillardia_theta_CCMP2712'
Viridiplantae
Chlorophyta
Chlamydomonadales
'Chlamydomonas_reinhardtii'
'Volvox_carteri_f._nagariensis'
'Micromonas_sp._RCC299'
'Chlorella_variabilis'
Embryophyta
Tracheophyta
Spermatophyta
'Picea_sitchensis'
Magnoliophyta
rosids
fabids
Malpighiales
Euphorbiaceae
Papilionoideae
Arabidopsis
'Populus_trichocarpa'
'Ricinus_communis'
'Jatropha_curcas'
'Glycine_max'
'Medicago_truncatula'
'Arabidopsis_thaliana'
'Arabidopsis_lyrata_subsp._lyrata'
'Vitis_vinifera'
Poaceae
Pooideae
Triticeae
Andropogoneae
'Hordeum_vulgare'
'Triticum_aestivum'
'Brachypodium_distachyon'
'Sorghum_bicolor'
'Zea_mays'
'Selaginella_moellendorffii'
'Physcomitrella_patens_subsp._patens'
Haptophyta
'Emiliania_huxleyi_CCMP1516'
SAR
stramenopiles
'Ectocarpus_siliculosus'
Oomycetes
Phytophthora
'Phytophthora_sojae'
'Phytophthora_infestans_T30-4'
'Albugo_laibachii_Nc14'
Alveolata
Apicomplexa
Aconoidasida
Plasmodium
Plasmodium_(plasmodium)
Piroplasmida
'Plasmodium_knowlesi_strain_H'
'Plasmodium_vivax_Sal-1'
'Plasmodium_chabaudi_chabaudi'
'Plasmodium_falciparum_3D7'
'Theileria_parva_strain_Muguga'
'Babesia_bovis_T2Bo'
Sarcocystidae
'Toxoplasma_gondii_ME49'
'Neospora_caninum_Liverpool'
Hymenostomatida
'Tetrahymena_thermophila'
'Ichthyophthirius_multifiliis'
'Bigelowiella_natans_CCMP2755'

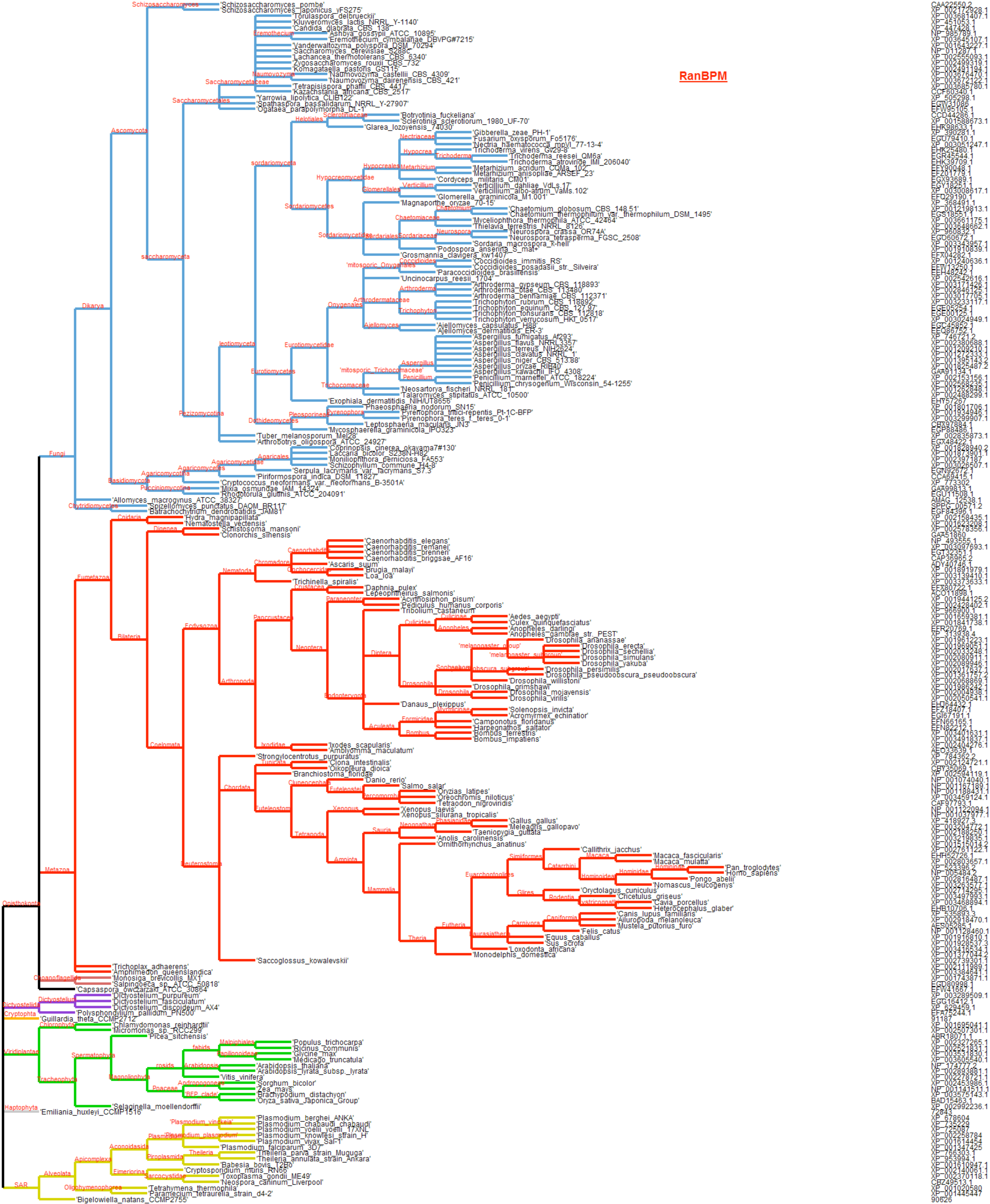

RanBPM
Fungi
Dikarya
Ascomycota
saccharomyceta
Saccharomycetales
Saccharomycetaceae
Schizosaccharomyces
'Schizosaccharomyces_pombe'
'Schizosaccharomyces_japonicus_yFS275'
'Torulaspora_delbrueckii'
'Kluyveromyces_lactis_NRRL_Y-1140'
'Candida_glabrata_CBS_138'
Eremothecium
'Ashbya_gossypii_ATCC_10895'
'Eremothecium_cymbalariae_DBVPG#7215'
'Vanderwaltozyma_polyspora_DSM_70294'
'Saccharomyces_cerevisiae_S288c'
'Lachancea_thermotolerans_CBS_6340'
'Zygosaccharomyces_rouxii_CBS_732'
'Komagataella_pastoris_GS115'
Naumovozyma
'Naumovozyma_castellii_CBS_4309'
'Naumovozyma_dairenensis_CBS_421'
'Tetrapisispora_phaffii_CBS_4417'
'Kazachstania_africana_CBS_2517'
'Yarrowia_lipolytica_CLIB122'
'Spathaspora_passalidarum_NRRL_Y-27907'
'Ogataea_parapolymorpha_DL-1'
Helotiales
Sclerotiniaceae
'Botryotinia_fuckeliana'
'Sclerotinia_sclerotiorum_1980_UF-70'
'Glarea_lozoyensis_74030'
sordariomyceta
Nectriaceae
'Gibberella_zeae_PH-1'
'Fusarium_oxysporum_Fo5176'
'Nectria_haematococca_mpVI_77-13-4'
Hypocrea
Trichoderma
'Trichoderma_virens_Gv29-8'
'Trichoderma_reesei_QM6a'
'Trichoderma_atroviride_IMI_206040'
Hypocreales
Metarhizium
'Metarhizium_acridum_CQMa_102'
'Metarhizium_anisopliae_ARSEF_23'
Hypocreomycetidae
'Cordyceps_militaris_CM01'
Glomerellales
Verticillium
'Verticillium_dahliae_VdLs.17'
'Verticillium_albo-atrum_VaMs.102'
'Glomerella_graminicola_M1.001'
Sordariomycetes
'Magnaporthe_oryzae_70-15'
Chaetomium
'Chaetomium_globosum_CBS_148.51'
'Chaetomium_thermophilum_var._thermophilum_DSM_1495'
Chaetomiaceae
'Myceliophthora_thermophila_ATCC_42464'
'Thielavia_terrestris_NRRL_8126'
Neurospora
'Neurospora_crassa_OR74A'
'Neurospora_tetrasperma_FGSC_2508'
Sordariomycetidae
Sordariales
Sordariaceae
'Sordaria_macrospora_k-hell'
'Podospora_anserina_S_mat+'
'Grosmannia_clavigera_kw1407'
'mitosporic_Onygenales'
Coccidioides
'Coccidioides_immitis_RS'
'Coccidioides_posadasii_str._Silveira'
'Paracoccidioides_brasiliensis'
'Uncinocarpus_reesii_1704'
Arthroderma
'Arthroderma_gypseum_CBS_118893'
'Arthroderma_otae_CBS_113480'
'Arthroderma_benhamiae_CBS_112371'
Onygenales
Arthrodermataceae
Trichophyton
'Trichophyton_rubrum_CBS_118892'
'Trichophyton_equinum_CBS_127.97'
'Trichophyton_tonsurans_CBS_112818'
'Trichophyton_verrucosum_HKI_0517'
Ajellomyces
'Ajellomyces_capsulatus_H88'
'Ajellomyces_dermatitidis_ER-3'
leotiomyceta
Eurotiomycetidae
'Aspergillus_fumigatus_Af293'
'Aspergillus_flavus_NRRL3357'
'Aspergillus_terreus_NIH2624'
'Aspergillus_clavatus_NRRL_1'
'Aspergillus_niger_CBS_513.88'
Aspergillus
'Aspergillus_oryzae_RIB40'
'Aspergillus_kawachii_IFO_4308'
Eurotiomycetes
'mitosporic_Trichocomaceae'
Penicillium
'Penicillium_marneffei_ATCC_18224'
'Penicillium_chrysogenum_Wisconsin_54-1255'
Trichocomaceae
'Neosartorya_fischeri_NRRL_181'
'Talaromyces_stipitatus_ATCC_10500'
'Exophiala_dermatitidis_NIH/UT8656'
Pezizomycotina
'Phaeosphaeria_nodorum_SN15'
Pleosporineae
Pyrenophora
'Pyrenophora_tritici-repentis_Pt-1C-BFP'
'Pyrenophora_teres_f._teres_0-1'
Dothideomycetes
'Leptosphaeria_maculans_JN3'
'Mycosphaerella_graminicola_IPO323'
'Tuber_melanosporum_Mel28'
'Arthrobotrys_oligospora_ATCC_24927'
Agaricales
'Coprinopsis_cinerea_okayama7#130'
'Laccaria_bicolor_S238N-H82'
Agaricomycetidae
'Moniliophthora_perniciosa_FA553'
Agaricomycetes
'Schizophyllum_commune_H4-8'
Agaricomycotina
'Serpula_lacrymans_var._lacrymans_S7.3'
'Piriformospora_indica_DSM_11827'
Basidiomycota
'Cryptococcus_neoformans_var._neoformans_B-3501A'
Pucciniomycotina
'Mixia_osmundae_IAM_14324'
'Rhodotorula_glutinis_ATCC_204091'
'Allomyces_macrogynus_ATCC_38327'
Chytridiomycetes
'Spizellomyces_punctatus_DAOM_BR117'
'Batrachochytrium_dendrobatidis_JAM81'
Cnidaria
'Hydra_magnipapillata'
'Nematostella_vectensis'
Digenea
'Schistosoma_mansoni'
'Clonorchis_sinensis'
'Caenorhabditis_elegans'
'Caenorhabditis_remanei'
Caenorhabditis
'Caenorhabditis_brenneri'
'Caenorhabditis_briggsae_AF16'
Chromadorea
'Ascaris_suum'
Onchocercidae
'Brugia_malayi'
Nematoda
'Loa_loa'
'Trichinella_spiralis'
Crustacea
'Daphnia_pulex'
'Lepeophtheirus_salmonis'
Paraneoptera
'Acyrthosiphon_pisum'
'Pediculus_humanus_corporis'
'Tribolium_castaneum'
Pancrustacea
Culicinae
'Aedes_aegypti'
Culicidae
'Culex_quinquefasciatus'
Anopheles
'Anopheles_darlingi'
'Anopheles_gambiae_str._PEST'
Ecdysozoa
Neoptera
'melanogaster_group'
'Drosophila_ananassae'
'Drosophila_erecta'
'Drosophila_sechellia'
'melanogaster_subgroup'
'Drosophila_simulans'
'Drosophila_yakuba'
Diptera
Sophophora
obscura_subgroup'
'Drosophila_persimilis'
'Drosophila_pseudoobscura_pseudoobscura'
Drosophila
'Drosophila_willistoni'
'Drosophila_grimshawi'
Arthropoda
Drosophila
'Drosophila_mojavensis'
'Drosophila_virilis'
Endopterygota
'Danaus_plexippus'
Myrmicinae
'Solenopsis_invicta'
'Acromyrmex_echinatior'
Formicidae
'Camponotus_floridanus'
Aculeata
'Harpegnathos_saltator'
Bombus
'Bombus_terrestris'
'Bombus_impatiens'
Coelomata
Ixodidae
'Ixodes_scapularis'
'Amblyomma_maculatum'
'Strongylocentrotus_purpuratus'
Tunicata
'Ciona_intestinalis'
'Oikopleura_dioica'
'Branchiostoma_floridae'
Chordata
'Danio_rerio'
Clupeocephala
'Salmo_salar'
Euteleostei
'Oryzias_latipes'
Percomorpha
'Oreochromis_niloticus'
'Tetraodon_nigroviridis'
Euteleostomi
Xenopus
'Xenopus_laevis'
'Xenopus_silurana_tropicalis'
Phasianidae
'Gallus_gallus'
Neognathae
'Meleagris_gallopavo'
Sauria
'Taeniopygia_guttata'
Tetrapoda
'Anolis_carolinensis'
'Ornithorhynchus_anatinus'
Amniota
Simiiformes
'Callithrix_jacchus'
Macaca
'Macaca_fascicularis'
'Macaca_mulatta'
Catarrhini
Homininae
'Pan_troglodytes'
'Homo_sapiens'
Hominidae
Hominoidea
'Pongo_abelii'
Deuterostomia
'Nomascus_leucogenys'
Euarchontoglires
Mammalia
'Oryctolagus_cuniculus'
Glires
Rodentia
'Cricetulus_griseus'
Hystricognathi
'Cavia_porcellus'
'Heterocephalus_glaber'
Caniformia
'Canis_lupus_familiaris'
'Ailuropoda_melanoleuca'
'Mustela_putorius_furo'
Eutheria
Carnivora
'Felis_catus'
Laurasiatheria
'Equus_caballus'
'Sus_scrofa'
Theria
'Loxodonta_africana'
'Monodelphis_domestica'
'Saccoglossus_kowalevskii'
Eumetazoa
Bilateria
Metazoa
'Trichoplax_adhaerens'
'Amphimedon_queenslandica'
Choanoflagellida
'Monosiga_brevicollis_MX1'
'Salpingoeca_sp._ATCC_50818'
Opisthokonta
'Capsaspora_owczarzaki_ATCC_30864'
Dictyostelium
'Dictyostelium_purpureum'
Dictyosteliida
'Dictyostelium_fasciculatum'
'Dictyostelium_discoideum_AX4'
'Polysphondylium_pallidum_PN500'
Cryptophyta
'Guillardia_theta_CCMP2712'
Chlorophyta
'Chlamydomonas_reinhardtii'
'Micromonas_sp._RCC299'
'Picea_sitchensis'
Malpighiales
'Populus_trichocarpa'
'Ricinus_communis'
fabids
Papilionoideae
'Glycine_max'
'Medicago_truncatula'
Viridiplantae
Spermatophyta
rosids
Arabidopsis
'Arabidopsis_thaliana'
'Arabidopsis_lyrata_subsp._lyrata'
Magnoliophyta
'Vitis_vinifera'
Andropogoneae
'Sorghum_bicolor'
Tracheophyta
Poaceae
'Zea_mays'
BEP_clade
'Brachypodium_distachyon'
'Oryza_sativa_Japonica_Group'
'Selaginella_moellendorffii'
Haptophyta
'Emiliania_huxleyi_CCMP1516'
'Plasmodium_berghei_ANKA'
'Plasmodium_(Vinckeia)'
'Plasmodium_chabaudi_chabaudi'
'Plasmodium_yoelii_yoelii_17XNL'
Plasmodium
'Plasmodium_(Plasmodium)'
'Plasmodium_knowlesi_strain_H'
'Plasmodium_vivax_Sal-1'
Aconoidasida
'Plasmodium_falciparum_3D7'
Theileria
'Theileria_parva_strain_Muguga'
Piroplasmida
'Theileria_annulata_strain_Ankara'
Apicomplexa
'Babesia_bovis_T2Bo'
Eimeriorina
'Cryptosporidium_muris_RN66'
Sarcocystidae
'Toxoplasma_gondii_ME49'
Alveolata
'Neospora_caninum_Liverpool'
SAR
Oligohymenophorea
'Tetrahymena_thermophila'
'Paramecium_tetraurelia_strain_d4-2'
'Bigelowiella_natans_CCMP2755'

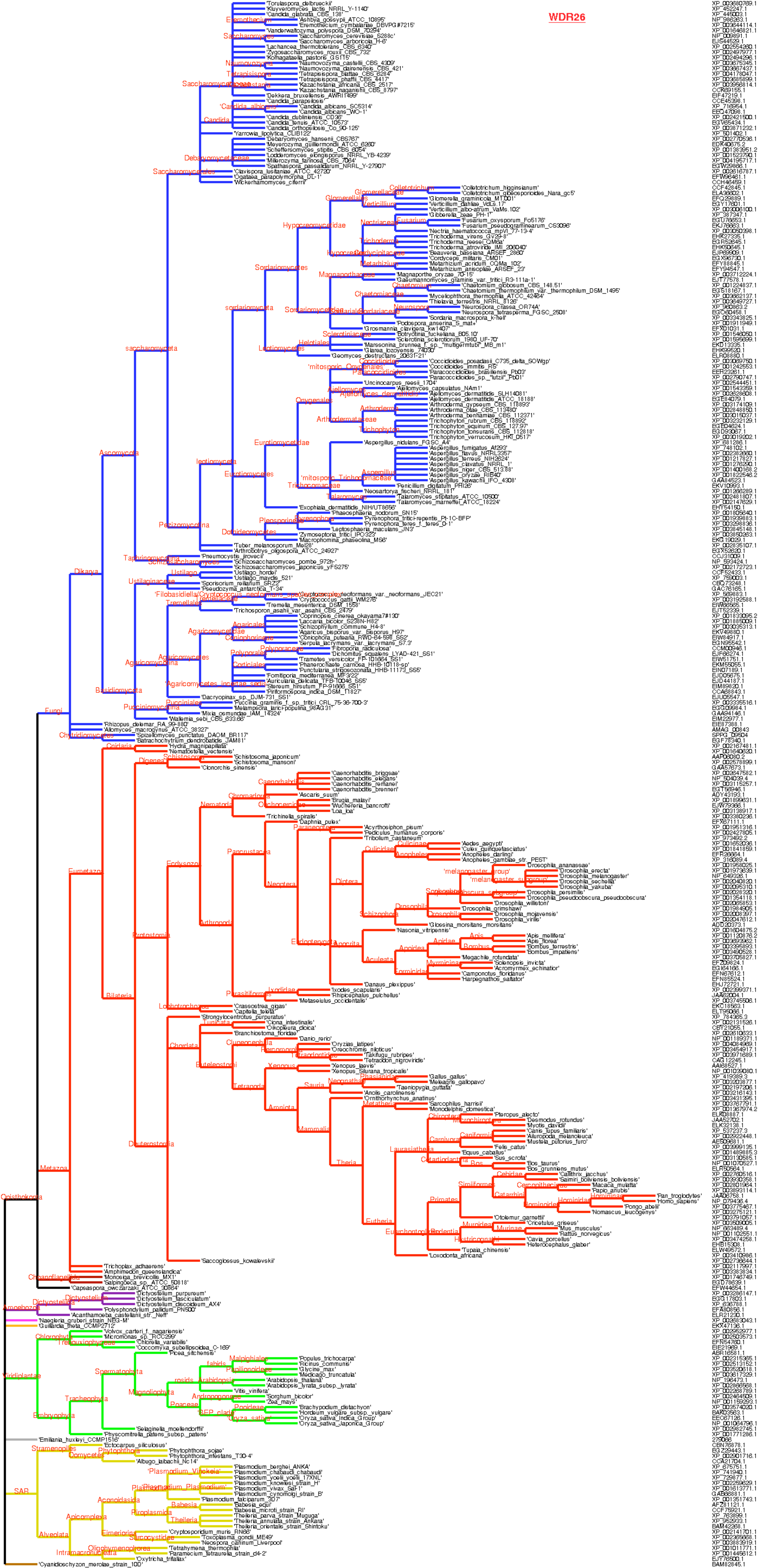
WDR26
Opisthokonta
Fungi
Dikarya
Ascomycota
Basidiomycota
Chytridiomycetes
Metazoa
Eumetazoa
Bilateria
Protostomia
Deuterostomia
Choanoflagellida
Amoebozoa
Viridiplantae
Stramenopiles
SAR
Alveolata
Apicomplexa

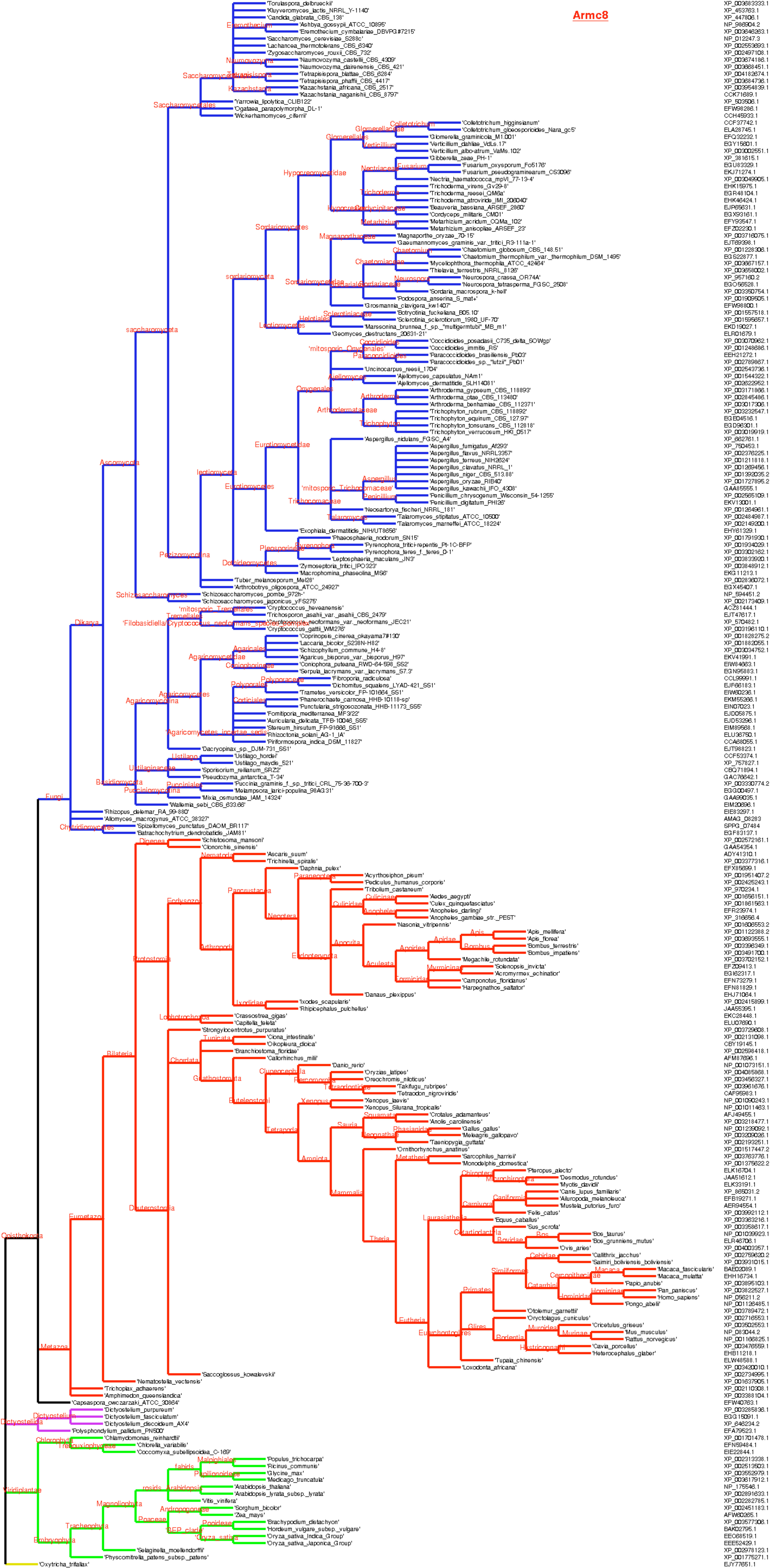
Armc8
'Torulaspora_delbrueckii' XP_003683333.1
'Kluyveromyces_lactis_NRRL_Y-1140' XP_453763.1
'Candida_glabrata_CBS_138' XP_447808.1
'Ashbya_gossypii_ATCC_10895' NP_986904.2
'Eremothecium_cymbalariae_DBVPG#7215' XP_003646263.1
'Saccharomyces_cerevisiae_S288c' NP_012247.3
'Lachancea_thermotolerans_CBS_6340' XP_002553893.1
'Zygosaccharomyces_rouxii_CBS_732' XP_002497108.1
'Naumovozyma_castellii_CBS_4309' XP_003674186.1
'Naumovozyma_dairenensis_CBS_421' XP_003668451.1
'Tetrapisispora_blattae_CBS_6284' XP_004182674.1
'Tetrapisispora_phaffii_CBS_4417' XP_003684736.1
'Kazachstania_africana_CBS_2517' XP_003954839.1
'Kazachstania_naganishii_CBS_8797' CCK71689.1
'Yarrowia_lipolytica_CLIB122' XP_503506.1
'Ogataea_parapolymorpha_DL-1' EFW98286.1
'Wickerhamomyces_ciferrii' CCH45933.1
'Colletotrichum_higginsianum' CCF37742.1
'Colletotrichum_gloeosporioides_Nara_gc5' ELA28745.1
'Glomerella_graminicola_M1.001' EFQ32232.1
'Verticillium_dahliae_VdLs.17' EGY15801.1
'Verticillium_albo-atrum_VaMs.102' XP_003002551.1
'Gibberella_zeae_PH-1' XP_381615.1
'Fusarium_oxysporum_Fo5176' EGU83329.1
'Fusarium_pseudograminearum_CS3096' EKJ71274.1
'Nectria_haematococca_mpVI_77-13-4' XP_003049905.1
'Trichoderma_virens_Gv29-8' EHK15975.1
'Trichoderma_reesei_QM6a' EGR48104.1
'Trichoderma_atroviride_IMI_206040' EHK46424.1
'Beauveria_bassiana_ARSEF_2860' EJP65631.1
'Cordyceps_militaris_CM01' EGX93161.1
'Metarhizium_acridum_CQMa_102' EFY93547.1
'Metarhizium_anisopliae_ARSEF_23' EFZ02230.1
'Magnaporthe_oryzae_70-15' XP_003716075.1
'Gaeumannomyces_graminis_var._tritici_R3-111a-1' EJT69398.1
'Chaetomium_globosum_CBS_148.51' XP_001228306.1
'Chaetomium_thermophilum_var._thermophilum_DSM_1495' EGS22877.1
'Myceliophthora_thermophila_ATCC_42464' XP_003657157.1
'Thielavia_terrestris_NRRL_8126' XP_003658002.1
'Neurospora_crassa_OR74A' XP_957160.2
'Neurospora_tetrasperma_FGSC_2508' EGO58528.1
'Sordaria_macrospora_k-hell' XP_003350754.1
'Podospora_anserina_S_mat+' XP_001909505.1
'Grosmannia_clavigera_kw1407' EFW98800.1
'Botryotinia_fuckeliana_B05.10' XP_001557518.1
'Sclerotinia_sclerotiorum_1980_UF-70' XP_001595657.1
'Marssonina_brunnea_f._sp._"multigermtubi"_MB_m1' EKD19027.1
'Geomyces_destructans_20631-21' ELR01679.1
'Coccidioides_posadasii_C735_delta_SOWgp' XP_003070962.1
'Coccidioides_immitis_RS' XP_001248686.1
'Paracoccidioides_brasiliensis_Pb03' EEH21272.1
'Paracoccidioides_sp._"lutzii"_Pb01' XP_002789867.1
'Uncinocarpus_reesii_1704' XP_002543736.1
'Ajellomyces_capsulatus_NAm1' XP_001544322.1
'Ajellomyces_dermatitidis_SLH14081' XP_002622952.1
'Arthroderma_gypseum_CBS_118893' XP_003171866.1
'Arthroderma_otae_CBS_113480' XP_002845486.1
'Arthroderma_benhamiae_CBS_112371' XP_003017306.1
'Trichophyton_rubrum_CBS_118892' XP_003232547.1
'Trichophyton_equinum_CBS_127.97' EGE04516.1
'Trichophyton_tonsurans_CBS_112818' EGD96301.1
'Trichophyton_verrucosum_HKI_0517' XP_003019919.1
'Aspergillus_nidulans_FGSC_A4' XP_662761.1
'Aspergillus_fumigatus_Af293' XP_750453.1
'Aspergillus_flavus_NRRL3357' XP_002378225.1
'Aspergillus_terreus_NIH2624' XP_001211818.1
'Aspergillus_clavatus_NRRL_1' XP_001269456.1
'Aspergillus_niger_CBS_513.88' XP_001392035.2
'Aspergillus_oryzae_RIB40' XP_001727895.2
'Aspergillus_kawachii_IFO_4308' GAA85555.1
'Penicillium_chrysogenum_Wisconsin_54-1255' XP_002565109.1
'Penicillium_digitatum_PHI26' EKV13001.1
'Neosartorya_fischeri_NRRL_181' XP_001264961.1
'Talaromyces_stipitatus_ATCC_10500' XP_002484987.1
'Talaromyces_marneffei_ATCC_18224' XP_002149200.1
'Exophiala_dermatitidis_NIH/UT8656' EHY61329.1
'Phaeosphaeria_nodorum_SN15' XP_001791930.1
'Pyrenophora_tritici-repentis_Pt-1C-BFP' XP_001934029.1
'Pyrenophora_teres_f._teres_0-1' XP_003302162.1
'Leptosphaeria_maculans_JN3' XP_003833920.1
'Zymoseptoria_tritici_IPO323' XP_003848912.1
'Macrophomina_phaseolina_MS6' EKG11213.1
'Tuber_melanosporum_Mel28' XP_002838072.1
'Arthrobotrys_oligospora_ATCC_24927' EGX45407.1
'Schizosaccharomyces_pombe_972h-' NP_594451.2
'Schizosaccharomyces_japonicus_yFS275' XP_002173409.1
'Cryptococcus_heveanensis' ACZ81444.1
'Trichosporon_asahii_var._asahii_CBS_2479' EJT47617.1
'Cryptococcus_neoformans_var._neoformans_JEC21' XP_570482.1
'Cryptococcus_gattii_WM276' XP_003196110.1
'Coprinopsis_cinerea_okayama7#130' XP_001828275.2
'Laccaria_bicolor_S238N-H82' XP_001882055.1
'Schizophyllum_commune_H4-8' XP_003034752.1
'Agaricus_bisporus_var._bisporus_H97' EKV41991.1
'Coniophora_puteana_RWD-64-598_SS2' EIW84663.1
'Serpula_lacrymans_var._lacrymans_S7.3' EGN95883.1
'Fibroporia_radiculosa' CCL99991.1
'Dichomitus_squalens_LYAD-421_SS1' EJF66183.1
'Trametes_versicolor_FP-101664_SS1' EIW60236.1
'Phanerochaete_carnosa_HHB-10118-sp' EKM55268.1
'Punctularia_strigosozonata_HHB-11173_SS5' EIN07023.1
'Fomitiporia_mediterranea_MF3/22' EJD05875.1
'Auricularia_delicata_TFB-10046_SS5' EJD53296.1
'Stereum_hirsutum_FP-91666_SS1' EIM89568.1
'Rhizoctonia_solani_AG-1_IA' ELU36750.1
'Piriformospora_indica_DSM_11827' CCA68055.1
'Dacryopinax_sp._DJM-731_SS1' EJT98823.1
'Ustilago_hordei' CCF53374.1
'Ustilago_maydis_521' XP_757827.1
'Sporisorium_reilianum_SRZ2' CBQ71894.1
'Pseudozyma_antarctica_T-34' GAC76842.1
'Puccinia_graminis_f._sp._tritici_CRL_75-36-700-3' XP_003330774.2
'Melampsora_larici-populina_98AG31' EGG00497.1
'Mixia_osmundae_IAM_14324' GAA99035.1
'Wallemia_sebi_CBS_633.66' EIM20896.1
'Rhizopus_delemar_RA_99-880' EIE83297.1
'Allomyces_macrogynus_ATCC_38327' AMAG_08283
'Spizellomyces_punctatus_DAOM_BR117' SPPG_07484
'Batrachochytrium_dendrobatidis_JAM81' EGF83137.1
'Schistosoma_mansoni' XP_002572161.1
'Clonorchis_sinensis' GAA54354.1
'Ascaris_suum' ADY41310.1
'Trichinella_spiralis' XP_003377316.1
'Daphnia_pulex' EFX85699.1
'Acyrthosiphon_pisum' XP_001951407.2
'Pediculus_humanus_corporis' XP_002425243.1
'Tribolium_castaneum' XP_970234.1
'Aedes_aegypti' XP_001656151.1
'Culex_quinquefasciatus' XP_001861563.1
'Anopheles_darlingi' EFR23974.1
'Anopheles_gambiae_str._PEST' XP_316656.4
'Nasonia_vitripennis' XP_001606553.2
'Apis_mellifera' XP_001122388.2
'Apis_florea' XP_003693555.1
'Bombus_terrestris' XP_003396349.1
'Bombus_impatiens' XP_003491700.1
'Megachile_rotundata' XP_003702152.1
'Solenopsis_invicta' EFZ09413.1
'Acromyrmex_echinatior' EGI62317.1
'Camponotus_floridanus' EFN73279.1
'Harpegnathos_saltator' EFN81829.1
'Danaus_plexippus' EHJ71084.1
'Ixodes_scapularis' XP_002415899.1
'Rhipicephalus_pulchellus' JAA55395.1
'Crassostrea_gigas' EKC28448.1
'Capitella_teleta' ELU07890.1
'Strongylocentrotus_purpuratus' XP_003729608.1
'Ciona_intestinalis' XP_002131098.1
'Oikopleura_dioica' CBY19145.1
'Branchiostoma_floridae' XP_002598418.1
'Callorhinchus_milii' AFM87896.1
'Danio_rerio' NP_001073151.1
'Oryzias_latipes' XP_004085868.1
'Oreochromis_niloticus' XP_003456327.1
'Takifugu_rubripes' XP_003961676.1
'Tetraodon_nigroviridis' CAF95963.1
'Xenopus_laevis' NP_001090243.1
'Xenopus_Silurana_tropicalis' NP_001011463.1
'Crotalus_adamanteus' AFJ49455.1
'Anolis_carolinensis' XP_003218477.1
'Gallus_gallus' NP_001239092.1
'Meleagris_gallopavo' XP_003209026.1
'Taeniopygia_guttata' XP_002193251.1
'Ornithorhynchus_anatinus' XP_001517447.2
'Sarcophilus_harrisii' XP_003763776.1
'Monodelphis_domestica' XP_001375622.2
'Pteropus_alecto' ELK16704.1
'Desmodus_rotundus' JAA51612.1
'Myotis_davidii' ELK33191.1
'Canis_lupus_familiaris' XP_865031.2
'Ailuropoda_melanoleuca' EFB19271.1
'Mustela_putorius_furo' AER94554.1
'Felis_catus' XP_003992112.1
'Equus_caballus' XP_003363216.1
'Sus_scrofa' XP_003358617.1
'Bos_taurus' NP_001039923.1
'Bos_grunniens_mutus' ELR46708.1
'Ovis_aries' XP_004003357.1
'Callithrix_jacchus' XP_002759620.2
'Saimiri_boliviensis_boliviensis' XP_003931015.1
'Macaca_fascicularis' BAE02089.1
'Macaca_mulatta' EHH16734.1
'Papio_anubis' XP_003895103.1
'Pan_paniscus' XP_003822527.1
'Homo_sapiens' NP_056211.2
'Pongo_abelii' NP_001128485.1
'Otolemur_garnettii' XP_003789472.1
'Oryctolagus_cuniculus' XP_002716553.1
'Cricetulus_griseus' XP_003502553.1
'Mus_musculus' NP_083044.2
'Rattus_norvegicus' NP_001166825.1
'Cavia_porcellus' XP_003476559.1
'Heterocephalus_glaber' EHB11218.1
'Tupaia_chinensis' ELW48588.1
'Loxodonta_africana' XP_003420010.1
'Saccoglossus_kowalevskii' XP_002734995.1
'Nematostella_vectensis' XP_001637905.1
'Trichoplax_adhaerens' XP_002110308.1
'Amphimedon_queenslandica' XP_003381004.1
'Capsaspora_owczarzaki_ATCC_30864' EFW40763.1
'Dictyostelium_purpureum' XP_003285836.1
'Dictyostelium_fasciculatum' EGG15091.1
'Dictyostelium_discoideum_AX4' XP_646234.2
'Polysphondylium_pallidum_PN500' EFA79523.1
'Chlamydomonas_reinhardtii' XP_001701478.1
'Chlorella_variabilis' EFN59484.1
'Coccomyxa_subellipsoidea_C-169' EIE22844.1
'Populus_trichocarpa' XP_002313338.1
'Ricinus_communis' XP_002513503.1
'Glycine_max' XP_003552979.1
'Medicago_truncatula' XP_003617912.1
'Arabidopsis_thaliana' NP_175546.1
'Arabidopsis_lyrata_subsp._lyrata' XP_002891633.1
'Vitis_vinifera' XP_002282785.1
'Sorghum_bicolor' XP_002451183.1
'Zea_mays' AFW80265.1
'Brachypodium_distachyon' XP_003577306.1
'Hordeum_vulgare_subsp._vulgare' BAK02795.1
'Oryza_sativa_Indica_Group' EEC68519.1
'Oryza_sativa_Japonica_Group' EEE52429.1
'Selaginella_moellendorffii' XP_002978123.1
'Physcomitrella_patens_subsp._patens' XP_001775271.1
'Oxytricha_trifallax' EJY77651.1

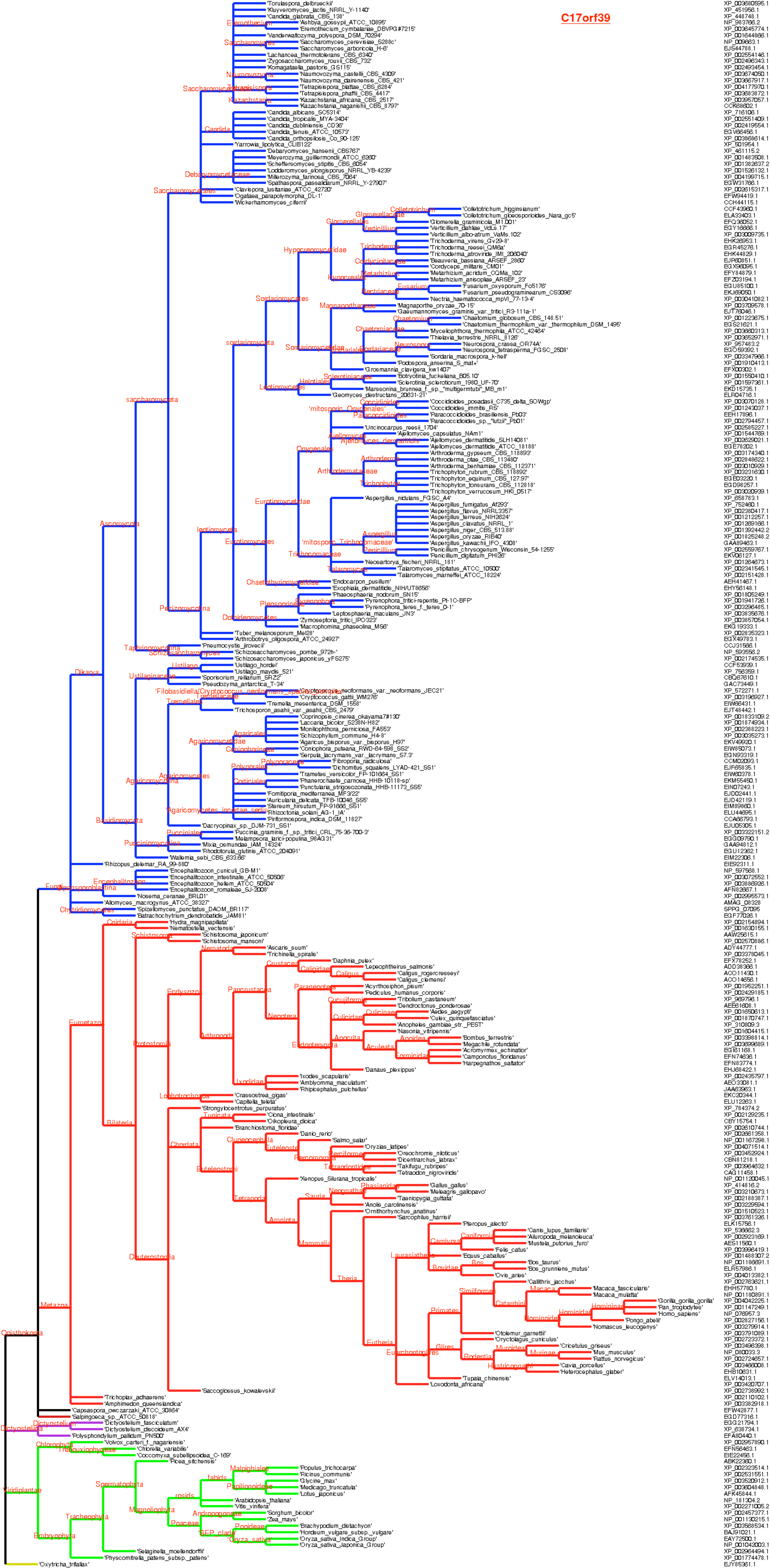
C17orf39
