# Supplementary material for: Molecular Phylogeny of a RING E3 Ubiquitin Ligase, Conserved in Eukaryotic Cells and Dominated by Homologous Components, the Muskelin/RanBPM/CTLH Complex
Source: PLoS One. 2013 Oct 15;8(10):e75217. doi: 10.1371/journal.pone.0075217 (PMC3797097; doi:10.1371/journal.pone.0075217)
Supplement: Figure S2 — The phylogenetic relationships of RanBPM, TWA1, MAEA and Rmnd5, based on their LisH/CTLH regions. 922 sequences spanning the LisH and CTLH regions were aligned as described in the Methods. The Newick output is presented here as a circular tree with proportionate branch lengths, with every entry identified by its GenBank accession number. Key: background shading denotes protein identities: blue = RanBPM, grey = TWA1, yellow = Rmnd5, pink = muskelin and green = MAEA. Colours of branches, text and outer band denote the taxonomical grouping of each species. Dark red = choanoflagellates, red = metazoa, blue = fungi, green = plants, brown = SAR, purple = amoebozoa, pink = excavate, black = independent opisthokont, orange = cryptophyte and dark grey = haptophyte, respectively. clustered by either algorithm; scale bar indicates substitutions/site. Branch support values are bootstrap values based on bootstop autoconvergence at 650 cycles. (PDF) [file pone.0075217.s002.pdf]

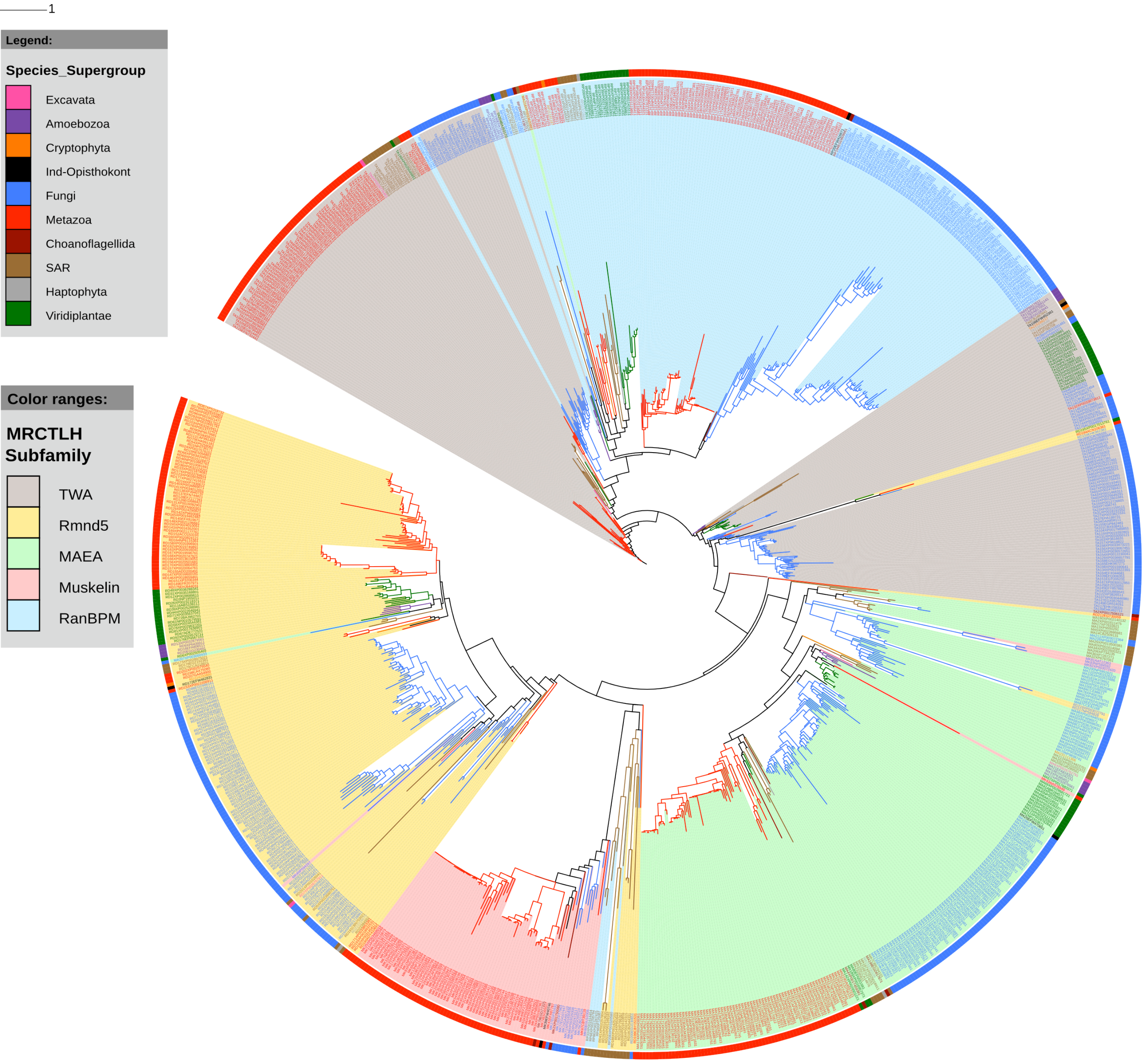

1
Legend:
Species_Supergroup
Excavata
Amoebozoa
Cryptophyta
Ind-Opisthokont
Fungi
Metazoa
Choanoflagellida
SAR
Haptophyta
Viridiplantae
Color ranges:
MRCTLH Subfamily
TWA
Rmnd5
MAEA
Muskelin
RanBPM
