# Supplementary figures and images for: Molecular Phylogeny of a RING E3 Ubiquitin Ligase, Conserved in Eukaryotic Cells and Dominated by Homologous Components, the Muskelin/RanBPM/CTLH Complex
Source: PLoS One. 2013 Oct 15;8(10):e75217. doi: 10.1371/journal.pone.0075217 (PMC3797097; doi:10.1371/journal.pone.0075217)

Figure S3

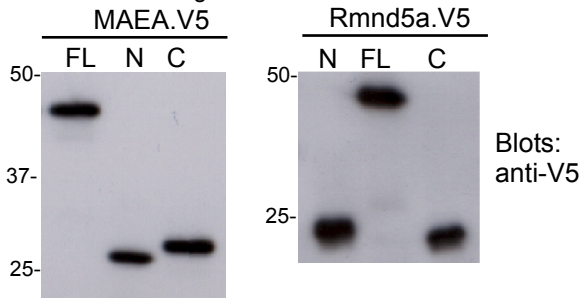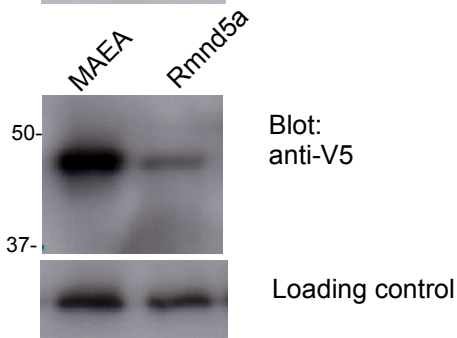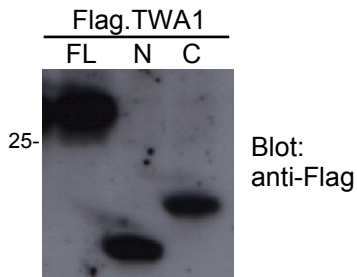

Supplement: Figure S3 — Expression of tagged MAEA, TWA1, Rmnd5a and their N- or C-terminal domain deletions. Proteins were transiently expressed by plasmid transfection into COS-7 cells as described in the Methods. After 48 h, whole cell lysates were prepared in SDS-PAGE sample buffer, resolved on 12.5% polyacrylamide gels under reducing conditions and transferred to PVDF membranes for immunoblotting with antibodies to the tags as indicated in the figure panels. All proteins had the expected apparent molecular masses. Protein levels of Rmnd5a were markedly lower than those of MAEA when compared on the same blot (middle panel). Immunoblot analysis of RanBP9 domains was published in [16]. FL = full-length; N = N-terminal domains, C = C-terminal domains as in Fig. 5. Molecular mass markers are given in kDa. (PDF) [file pone.0075217.s003.pdf]
