# Supplementary material for: Molecular Phylogeny of a RING E3 Ubiquitin Ligase, Conserved in Eukaryotic Cells and Dominated by Homologous Components, the Muskelin/RanBPM/CTLH Complex
Source: PLoS One. 2013 Oct 15;8(10):e75217. doi: 10.1371/journal.pone.0075217 (PMC3797097; doi:10.1371/journal.pone.0075217)
Supplement: Table S1 — Oligonucleotides used for preparation of expression constructs. (PDF) [file pone.0075217.s004.pdf]

Table S1. Oligonucleotides used for preparation of mammalian expression constructs for this study

Oligonucleotide	Sequence, 5' to 3'
Maea 501F	GGGATCTTCAAGATGGCG
Maea 502R	CATGATGTACACCTTCTC
Maea N-terminus 640R	AGCGTCCAGTCTCTTATTCTG
Maea C-terminus 641F	GGCCGCTTCAAGATGTTCCTGACGGCCAAAGAGGTG
Rmnd5a 503F	GCCGTCTCCGGCATGGAT
Rmnd5a 504R	GAAAAATATCTGTTTGGC
Rmnd5a N-terminus 648R	ATTTGTGGTTCCACCCATTAA
Rmnd5a C-terminus 650F	GGCCGCTTCAAGATGCCATTTGTGGAGTTAAATAG
TWA1 267F	CTAGAAGCTTATGAGTTATGCAGAAAAG
TWA1 268R	GATCGAATCGCTCTACTTGGGCTCCTC
TWA1 N-terminus 643R	CGATGAATTCCTATGCTTCTGTCTCACG
TWA1 C-terminus 642F	CTAGAAGCTTACACTTGATGAGCGAATC
